# Phenotypic characterization of a dominant glossy mutant and fine mapping of *qCWA9.1* for controlling cuticular wax biosynthesis in rapeseed (*Brassica napus* L.)

**DOI:** 10.3389/fpls.2026.1897630

**Published:** 2026-07-14

**Authors:** Lei Lei, Xirong Zhou, Weirong Wang, Xianmin Meng, Hongru Liu, Hongwei Li, Jifeng Zhu

**Affiliations:** 1 Key Laboratory of Germplasm Innovation and Genetic Improvement of Grain and Oil Crops (Co-construction by Ministry and Province), Ministry of Agriculture and Rural Affairs, Shanghai, China; 2 Key Laboratory of Agricultural Genetics and Breeding of Shanghai, Crop Breeding and Cultivation Research Institute, Shanghai Academy of Agricultural Sciences, Shanghai, China

**Keywords:** cuticular wax, *CYP86A4*, cytochrome P450 family, QTL mapping, rapeseed

## Abstract

**Introduction:**

Plant cuticular wax has gained increasing attention owing to its critical role in biotic and abiotic stress tolerance. Identifying key genes regulating cuticular wax biosynthesis and understanding their mechanisms are important for rapeseed resistance breeding. However, the candidate genes and mechanisms involved in cuticular wax biosynthesis in rapeseed remain poorly understood.

**Methods and results:**

In this study, a dominant glossy mutant *39J7H* was identified in rapeseed through genetic analysis. Two stable and pure sister lines ‘4074’ (glaucous) and ‘4075’ (glossy) were developed from the glossy mutant *39J7H* via successive self-crosses. The leaves of ‘4075’ exhibited significantly increased cuticle permeability as indicated by water loss rate and toluidine blue (TB) staining. Conversely, scanning electron microscopy (SEM) and GC-MS analysis demonstrated a dramatic reduction in wax crystals density, total wax content, and chemical components in ‘4075’ leaves compared to ‘4074’. Gene mapping analysis using F_2_ populations from the ‘4074’ × ‘4075’ cross identified the *qCWA9.1* locus within a 2.3 Mb region on chromosome A09 of rapeseed through BSA-seq. Subsequent linkage analysis in the F_2_ population, along with phenotypic data from the F_2:3_ generation, refined the *qCWA9.1* region to a 49.0-kb interval between the SSR markers 9AS337 and 9AS339. This interval contains nine genes, among which *BnaA09G0695600ZS*, an orthologue of the Arabidopsis gene *AT1G01600* (*AtCYP86A4*), encoding a fatty acid omega-hydroxylase *CYP86A4*, was identified as a potential candidate gene for *qCWA9.1* associated with the glossy phenotype. Sequence analysis uncovered SNP variations in the first exon and promoter region of *BnaA09G0695600ZS* in the glossy line ‘4075’. Additionally, qRT-PCR analysis indicated that a significant upregulated in *BnaA09G0695600ZS* expression in this line.

**Discussion:**

Based on these results, we hypothesize that *BnaA09G0695600ZS* emerged as one of candidate genes for cuticular wax synthesis. The absence of two key cis-regulatory elements, I-box and TATC-box, in the promoter region of *BnaA09G0695600ZS* in the glossy line ‘4075’ may potentially alter cuticular wax metabolism in rapeseed leaves. These findings enhance our understanding of cuticular wax biosynthesis in rapeseed and provide valuable genetic resources for the development of high-stress-resistant rapeseed germplasms.

## 1 Introduction

Rapeseed (*Brassica napus* L.), as the third most important oil crop worldwide, is valued for its high oil content and exemplary agronomic traits, thus providing a reliable source of premium edible oils and industrial raw materials. However, its utilization is limited by various biotic and abiotic stressors. The cuticle serves as a thin hydrophobic layer covering the surface of terrestrial plants as well as a protective barrier against environmental stress. Although research on cuticle formation and function in rapeseed is limited, it has been well documented in *Arabidopsis thaliana*. This cuticle, covering the aerial parts of plants, is crucial for the survival and development of plants in terrestrial environments (Bach et al., 2008; Chen et al., 2023; Kong et al., 2020; Lee et al., 2020; Xu et al., 2021). The cuticle can protect plants from non-stomatal water loss and serves as a barrier against various biotic and abiotic stresses, including drought, low temperatures, UV radiation, insect pests, and pathogens (González-Valenzuela et al., 2023; Wang and Chang, 2022; Zhao et al., 2026a; Zhu et al., 2022).

The plant cuticle is a complex structure consisting of two main components: cutin and cuticular wax (Bernard and Joubès, 2013; Lee et al., 2020; Suh et al., 2005). Cutin is an insoluble polyester primarily composed of hydroxy and epoxy fatty acids, diacids, and glycerol, forming a backbone of the cuticle (Pollard et al., 2008; Li-Beisson et al., 2013; Fich et al., 2016). To date, the structure of cutin polymers remains largely unknown; however, genetic studies on *Arabidopsis thaliana* have identified numerous genes involved in cutin monomer biosynthesis (Fich et al., 2016). These genes play distinct roles in monomer biosynthesis, transport, export, and polymerization pathways (**Supplementary Figure 1**). Cutin monomers are synthesized intracellularly through cytochrome P450 (CYP)-dependent hydroxylation and epoxidation reactions (Kolattukudy, 2001; Liu et al., 2025; Pinot and Beisson, 2011). Common cutin monomers typically include C16 and C18 ω-hydroxy fatty acids, which feature a terminal hydroxyl (ω-OH) as well as one or more mid-chain oxygenations (such as hydroxy or epoxy groups). Examples include 10,16-dihydroxyhexadecanoic acid and 18-hydroxy-9,10-epoxyoctadecanoic acid (Pollard et al., 2008; Fich et al., 2016).

Cuticular waxes are aliphatic mixture primarily composed of very-long-chain fatty acids (VLCFAs) and their derivatives, including alcohols, alkanes, aldehydes, ketones, and wax esters (Bernard and Joubès, 2013; Lee and Suh, 2015; Huang et al., 2023). These compounds typically have chain lengths ranging from 20 to 36 carbon atoms. Enzymes from the CYP77A, CYP86A, CYP94B, and CYP704 subfamilies of the P450 family are known to catalyze hydroxylation either at the mid-chain positions or at the terminal methyl group (ω-hydroxylation) of aliphatic fatty acid chains (**Supplementary Figure 1**). These waxes are either embedded in the cutin matrix or are deposited on the plant surface (Yeats and Rose, 2013; Wan et al., 2022; Erndwein et al., 2023). Long-chain fatty acids (C16–C18) are synthesized in the plastids by fatty acid synthases, esterified by the fatty acid elongase (FAE) complex, and transported to the endoplasmic reticulum (ER) to form VLCFA-CoAs (**Supplementary Figure 1**). VLCFA-CoAs are modified by either the acyl-reduction pathway, which produces even-numbered primary alcohols and alkyl esters, or the decarbonylation pathway, which yields even-numbered aldehydes, secondary alcohols, odd-numbered alkanes, and ketones (**Supplementary Figure 1**). Key genes in *Arabidopsis*, including *CER1*, *CER3*, *CER4*, *CER6*, *CER10*, *CYTB5*, *KCR1*, *MAH1*, and *PAS2* are essential for cuticular wax biosynthesis (Bernard and Joubès, 2013; Fukuda et al., 2022; Niu et al., 2026).

Cytochrome P450 is one of the largest families of enzymes in plants and plays a crucial role in various growth and developmental processes, including cuticle synthesis. Fatty acid hydroxylases play a crucial role in the biosynthesis of the plant cuticle. *CYP86A1*, *CYP86A2*, *CYP86A4*, *CYP86A7*, and *CYP86A8* have been reported to function as fatty acid ω-hydroxylases involved in cutin synthesis in *Arabidopsis* (Höfer et al., 2008; Wellesen et al., 2001; Xiao et al., 2004). For instance, T-DNA insertional mutants (*cyp86a4–1* and *cyp86a4-2*) of *AtCYP86A4* exhibited a 45–58% reduction in the levels of 16-hydroxypalmitate, 10,16-dihydroxypalmitate, and 1,16-hexadecanedioic acid in flowers. This confirmed that AtCYP86A4 is capable of hydroxylating C16:0 fatty acids to produce C16-ω-OH fatty acids in the 10,16-dihydroxypalmitate biosynthetic pathway (**Supplementary Figure 1i**) (Li-Beisson et al., 2009).

The latest research reported that the overexpression of *PtrCYP86A7*/*PtrCYP86A8* is also involved in cuticular wax biosynthesis in poplar (Zhao et al., 2026a). CYP704B1 and CYP704B2 can catalyze the ω-hydroxylation of long-chain fatty acids in *Arabidopsis* and rice pollen and participate in the synthesis of sporopollenin and pheromones, respectively (Dobritsa et al., 2009; Li et al., 2010). P450 enzymes also contribute to wax synthesis, with *Arabidopsis* CYP96A15 functioning as a mid-chain alkane hydroxylase (MAH1), facilitating the conversion of alkanes to secondary alcohols and ketones, which are key components of cuticular wax (Greer et al., 2007). Additionally, CYP96B5 is involved in the catalysis of alkane hydroxylation to form odd-numbered primary alcohols, contributing to the formation of epidermal wax crystals in rice leaves (Zhang et al., 2020). Recent research suggests that CYP96A4 promotes the biosynthesis of cuticular waxes and cutin monomers via the jasmonic acid (JA) signaling pathway in *Arabidopsis* (Huang et al., 2024). All the aforementioned proteins belong to four subfamilies, CYP86, CYP94, CYP96, and CYP704, within the CYP86 clan of the cytochrome P450 family. Members of these subfamilies can be distinguished by expression patterns, substrate specificity, and region selectivity (Duan and Schuler, 2005; Pinot and Beisson, 2011; Wellesen et al., 2001). For example, AtCYP86A8 catalyzes the ω-hydroxylation of C12 to C18:1 fatty acid, demonstrating the highest activity towards palmitic and oleic acids, whereas CYP94 family enzymes are more effective at hydroxylating 9,10-epoxystearate (Pinot et al., 1999; Pinot and Beisson, 2011).

Despite much progress in elucidating the molecular mechanisms underlying cuticular wax biosynthesis in crops such as *Arabidopsis* and rice, there remains a paucity of knowledge regarding the genetic control of cuticular wax synthesis in the allotetraploid species *Brassica napus*, a close relative of *Arabidopsis* and a member of the Brassicaceae family. In *Brassica napus*, only a small number of genes involved in cuticular wax synthesis have been identified. In 2013, a dominant glossy mutant (*BnaA.GL*) was reported and genetic analysis was conducted (Pu et al., 2013). *BnaCER1*, located on chromosome A09, is the candidate gene responsible for cuticular wax synthesis, based on genetic mapping and transcriptional expression. This study revealed an 89% reduction in total cuticular wax and altered the wax composition in the *GL* mutant. These findings indicate that the glossy phenotype is closely associated with the level and composition of cuticular wax. Subsequently, it has been reported that overexpression of *BnKCS1-1*, *BnKCS1-2*, and *BnCER1–2* led to a significant increase in cuticular wax on transgenic *Brassica napus* leaves (Wang et al., 2020). *BnaA1.CER4* and *BnaC1.CER4* were identified as encoding fatty acyl-coenzyme A reductases, which regulate the synthesis of anteiso-primary alcohols (Liu et al., 2021). Recently, several members of the P450 family, including *CYP71B2* (*BnaC04g35220D*), *CYP96A8* (*BnaA08g16470D*), *CYP86A4* (*BnaA10g00380D*), and *CYP96A15* (*BnaC09g51620D*), have been proposed as candidate genes for cuticular wax biosynthesis in *Brassica napus* leaves, based on genome-wide association study (GWAS) and transcriptomic data (Jin et al., 2020; Long et al., 2023).

Natural genetic variation in plants has been instrumental in identifying key genes that govern complex agronomic traits such as growth, flowering, and stress tolerance (Weigel, 2012; Moore et al., 2020). Consequently, it is crucial to dissect the genetic basis of cuticular wax synthesis and identify candidate genes using natural field variation. The objective of this study was to identify candidate genes associated with cuticular wax synthesis by using a novel dominant glossy mutant, *39J7H*, in rapeseed with natural variation. In this study, we identified a major quantitative trait locus (QTL) controlling cuticular wax synthesis in ChrA09 using a bulked segregant analysis sequencing (BSA-seq) approach. This QTL was further refined to a 49-Kb interval by fine mapping, and the candidate gene for this locus was predicted via sequence comparisons and expression analysis. The gene *BnaA09G0695600ZS*, annotated as a member of the CYP86A subfamily of cytochrome P450 genes and encoding fatty acid omega-hydroxylase, was proposed as the candidate gene involved in cuticular wax synthesis. The findings presented here lay a foundation for further studies on the genetic architecture and molecular mechanisms underlying cuticular wax synthesis in rapeseed and provide valuable genetic resources for improving the resistance of rapeseed germplasm to biotic and abiotic stresses.

## 2 Materials and methods

### 2.1 Plant materials

A glossy mutant line *39J7H* was discovered in rapeseed under natural field conditions at the research base of the Shanghai Academy of Agricultural Sciences (30.88°N, 121.38°E, Shanghai, China). Phenotypic segregation occurred in the self-crossed offspring of mutant *39J7H*. Three glossy and glaucous plants were selected from the offspring and continuously self-crossed until stable, pure sister lines were obtained: the glaucous line ‘4074’ and the glossy line ‘4075’. The F_1_ plants from the cross ‘4074’ ×’4075’ were all glossy, and an F_2_ segregating population was generated through self-crossing and grown under natural field conditions. Both the ‘4074’ and ‘4075’ lines belong to the winter-type rapeseed, which is usually sown in mid-October each year in Shanghai. All the plant materials were cultivated at the Shanghai Academy of Agricultural Sciences Research Base.

### 2.2 Scanning electron microscopy observation

The fourth true leaf from the top of 6-week-old rapeseed seedling was used for SEM observation. The dried leaf segments were sputter-coated with gold for 60 s using an SD-900 sputter coater (VPI, Beijing, China) and examined using a TM4000Plus tabletop microscope (Hitachi, Tokyo, Japan).

### 2.3 Toluidine blue assay

The toluidine blue (TB) assay assesses water permeability caused by surface defects, as described previously (Pu et al., 2013). Leaves at the same position of ‘4074’ and ‘4075’ seedling were detached and soaked in a 0.05% (w/v) toluidine blue solution for 10 minutes and subsequently rinsed with water to remove excess TB from the leaf surface.

### 2.4 Cuticle permeability analysis

To test the water loss rate, leaves at the same position of rapeseed plants were detached and placed in the dark for 12 hours to ensure stomatal closure. Then, weights were measured at different time points (0.5 h, 1 h, 1.5 h, 2 h, 3 h, 4 h, 5 h, 6 h, and 7 h) by a microbalance. The water loss rate was calculated as the percentage of fresh weight based on the initial weight.

### 2.5 Leaf cuticular wax measurement

The cuticular wax composition of leaves from the parental lines ‘4074’ and ‘4075’ were determined as described by Liu et al. (2021), with some modifications. Leaf sections (~10 cm^2^) were immersed in 10 mL chloroform (CHCl_3_) containing 10 μg n-tetracosane (C24) (Solarbio, Beijing, China) as an internal standard for 30 s at room temperature. After wax extraction, the leaves were photographed to measure the surface area using ImageJ software. The extracts were dried under a nitrogen stream and derivatized with 50 μL pyridine (Vokai, Beijing, China) for 30 min at 50 °C, followed by 50 μL N, O–bis (trimethylsilyl) trifluoroacetamide (BSTFA) (Solarbio, Beijing, China) for 40 min at 60 °C. The derivatized extract was dried again under a nitrogen stream and re-dissolved in 500 μL of chloroform for qualitative and quantitative wax analyses.

GC-MS analysis was performed using an Rtx-5 MS fused silica column with helium as the carrier gas at a flow rate of 1 mL/min in an Agilent 5977 inert MSD system. The analysis was conducted in electron impact ionization mode (70 eV, m/z 35 to 765) under constant velocity mode (4.2 min solvent delay). The injector and MSD interface temperatures were both set to 280 °C, and the ion source temperature was set to 250 °C. The GC program was based on previous reports (Liu et al., 2021) with some modifications. Briefly, the initial oven temperature of 50 °C for 2 minutes, followed by an increase of 5 °C/min to 240 °C, a 2 min hold at 240 °C, an increase of 10 °C/min to 320 °C, and a 3 min hold at 320 °C. The identification of the compounds was conducted through a comparative analysis of the mass spectra with those of the National Institute of Standards and Technology (NIST08). The quantification of each compound was determined by the calculation of peak areas relative to the internal standard tetracosane. The total wax amount per unit leaf area was expressed as μg·cm^−2^. Values represent the means of three biological replicates (mean ± SD).

### 2.6 BSA−seq linkage analysis

A total of 199 F_2_ individuals from the ‘4074’ × ‘4075’ crosses were used for mapping purposes. Fifty individuals each from the F_2_ population were selected to form the ‘1’ and ‘2’ bulks, based on the glaucous and glossy phenotypes. Genomic DNA was extracted from fresh leaves of the parents and F_2_ individuals using a plant genomic DNA extraction kit (ZOMANBIO, ZP309) via the CTAB method. Genomic DNA from the parents and two bulk pools were fragmented using sonication, followed by library construction with the TruSeq DNA PCR-free prep kit (Illumina, 20015963). The parents, along with the ‘1’ and ‘2’ bulks, underwent paired-end 150 bp whole-genome resequencing on the Illumina NovaSeq6000 platform (Illumina, Inc., San Diego, CA, USA). The raw data is saved in paired-end FASTQ format and the quality of the bases is assessed by their quality (Q-value). The quality of the sequencing data was assessed using Fastp (v0.23.1) and visualized using the R package. The raw data was further filtered using fastp (v0.20.0) to produce high quality data. The bwa (0.7.12-r1039) mem program was used to align the high-quality data obtained after filtering to the reference genome *Brassica napus* ‘ZS11’ v0 (http://yanglab.hzau.edu.cn/BnIR), and the parameters of the alignment were based on the default parameters of bwamem. The sam files were sorted and converted into bam files using picard 1.107 software and the “FixMateInformation” command was used to ensure the consistency of all paired-end reads information. Variant calling was implemented with GATK4 (GenomeAnalysisTK v3.8). Alignments near InDel are usually unreliable and need to be realigned using known InDel information via the RealignerTargetCreator and IndelRealigner command in the GATK package. SNP loci for the samples were obtained using the UnifiedGenotyper program with stand_call_conf set to 30 and stand_emit_conf set to 10. Finally, The SNP and INDEL loci were annotated using ANNOVAR software.

Quantitative trait locus (QTL) analysis was conducted using the SNP index method (Takagi et al., 2013), the Euclidean distance (ED) algorithm (Hill et al., 2013), and the G’-value method (Magwene et al., 2011). For SNP-index mapping analysis, the SNP-index value of each pool was calculated by custom Python scripts. ΔSNP-index was defined as: ΔSNP­index=SNP­index(glossy pool)−SNP­index(glaucous pool). A 1 Mb sliding window with 100 kb step size was used to smooth the ΔSNP-index distribution across all chromosomes. Regions with significantly elevated ΔSNP-index values were identified as candidate intervals controlling the leaf glossy trait, and visualized using R v4.2.1 ggplot2 package. SNP loci exhibiting genotype differences between the two mixed pools were utilized to statistically analyze the depth of each base in the different mixed pools, and the Euclidean Distance (ED) value for each locus was calculated. The ED values calculated using the formula: 


$$\text{ED}=\sqrt{{\left(\text{A}glossy-\text{A}glaucous\right)}^{2}+{\left(\text{C}glossy-\text{C}glaucous\right)}^{2}+{\left(\text{G}glossy-\text{G}glaucous\right)}^{2}+{\left(\text{T}glossy-\text{T}glaucous\right)}^{2}}$$

(ATCG)*glossy* indicates the frequency of A, T, C, and G base in the glossy pool, where (ATCG)*glaucous* represents the frequency of A, T, C, and G base in the glaucous pool. To eliminate the background noise caused by ED values with small differences, calculate ED^4 (the 4th power of the ED value) and perform LoessFit fitting on ED^4. Select ‘the median +3×standard deviation’ as the threshold. For the G’ value method, the G’ value was calculated using the QTLseqr package in R language to screen SNPs, and then the G-statistical value analysis was carried out using the runGprimeAnalysis function in the QTLseqr package with WINDOW GPRIME as the window.

### 2.7 Development of markers, genetic map construction, and fine mapping

The candidate region for the cuticular wax gene identified by BSA-seq was further refine by fine mapping using SSR markers. Interval sequences were downloaded from the *Brassica napus* multi-omics information resource database (https://yanglab.hzau.edu.cn/BnIR). For genotyping and fine mapping, 162 SSR markers were developed based on the interval sequences of 62.7 Mb-65.0 Mb. SSR primers were designed using Primer 5.0. The polymorphism of 162 SSR markers were screened via polyacrylamide gel electrophoresis between the two parents. Subsequently, the 11 SSR markers showed stable amplification and polymorphisms between the two parents, and these markers were used to genotype the F_2_ individuals of ‘4074’ × ‘4075’ population. The sequences of primers used for mapping are listed in **Supplementary Table 5**. A phenotypic marker G was obtained by investigating the phenotype of the F_2:3_ population. Combining the phenotypic data (glossy/wild-type leaf) and genotypic data of all recombinant lines, the recombination breakpoints of each exchange individual were determined. The target gene was localized between the two flanking markers where no further recombinants were detected, thus narrowing the candidate physical interval to a smaller genomic segment by constructing a genetic linkage map using MAP functionality with the map distance (cM) in QTL IciMapping v4.1 (Meng et al., 2015). After delimiting the minimal candidate region, all predicted protein-coding genes within the narrowed interval were extracted from the *Brassica napus* reference genome ‘ZS11’ v0. Gene functional annotation information was retrieved to screen candidate genes.

### 2.8 Gene prediction, cloning and sequence analyses of candidate genes

The SNP/InDels variations in genes within the physical intervals of the major QTL *qCWA9.1* were detected using GATKv3.8 (Zhu et al., 2015). The effects of the identified SNPs/InDels, including frameshift deletions, frameshift insertions, stopgains, stoplosses, and synonymous mutations, were assessed using ANNOVAR (https://annovar.openbioinformatics.org/en/latest/) (Wang et al., 2010) in a gene-based annotation model with default settings. The physical intervals of the major QTL *qCWA9.1* were aligned to the *Brassica napus* ‘ZS11’ reference genome to identify the corresponding annotated genes.

Nine annotated genes within the *qCWA9.1* interval were screened and cloned. The genomic DNA was extracted from the cotyledons of the two parental lines (‘4074’ and ‘4075’) using a modified CTAB protocol, followed by PCR amplification and genotyping. The PCR reaction mixture, with a total volume of 15 μL, consisted of 1 μL genomic DNA, 0.5 μL each of forward and reverse primers, 7.5 μL of 2× high-fidelity enzyme mix (Vazyme, Nanjing, China), and 5.5 μL ddH_2_O. The PCR program included an initial step at 94 °C for 3 min, followed by 35 cycles of 94 °C for 30 s, 55 °C for 30 s, and 72 °C for 150 s, 72 °C for 5 min, and a final hold at 4 °C. The PCR products were separated by 1% (w/v) agarose gel electrophoresis and sequenced by Sangon Biotech (Shanghai, China). The gene sequences of ‘4074’ and ‘4075’ were aligned using DNAMAN version 6.0 (Lynnon Biosoft, USA). A 2 kb fragment upstream of the ATG start codon of *BnaCYP86A4* (*BnaA09G0695600ZS*) was amplified from genomic DNA, and the cis-elements in the *BnaCYP86A4* promoter region were predicted using the PlantCARE website (http://bioinformatics.psb.ugent.be/webtools/plantcare/html/) (Lescot et al., 2002) and visualized using TBtools (Chen et al., 2020). The primers used for RT-PCR are listed in **Supplementary Table 5**.

### 2.9 RNA isolation and real-time quantitative PCR assay

Total RNA was extracted from the leaves of ‘4074’ and ‘4075’ using the RNAprep Pure Plant Kit (DP432, TianGen, Beijing, China) according to the manufacturer’s instructions. Reverse transcription was performed with 1 μg of total RNA using a TransScript^®^ All-in-One First-Strand cDNA Synthesis Kit (AT341; TransGen, Beijing, China). RT-qPCR was performed using the Perfect Start Green qPCR Super Mix Kit (AQ601-04, Transgen) on a QuantStudioTM 6 and 7 Flex Real-Time PCR System (Thermo Fisher Scientific, USA). Gene expression levels were calculated using the comparative Ct method with *BnaACTIN7* (*BnaC02G0037200ZS*) as the reference gene for data normalization (Liu et al., 2021). The primers used for RT-qPCR are listed in **Supplementary Table 6**. Three biological replicates were performed for each gene with three technical replicates per experiment.

### 2.10 Conserved domain, phylogenetic and three-dimensional protein structure prediction

Full-length BnaCYP86A4 (BnaA09G0695600ZS) proteins were used as queries to search for the closest relatives of *Brassica napus*, *Arabidopsis*, rice, and wheat using BLAST in the *Brassica napus* multi-omics information resource database (https://yanglab.hzau.edu.cn/BnIR), rice gene index (https://riceome.hzau.edu.cn) (Yu et al., 2023), and Phytozome database (https://phytozome-next.jgi.doe.gov/) (Goodstein et al., 2012). The retrieved peptide sequences were aligned using the ClustalW algorithm in MEGA7.0. A phylogenetic tree was then constructed using the maximum likelihood method with a Poisson model and 1,000 bootstrap replications and edited with FigTree v1.4.4. The conserved domains of proteins encoded by candidate genes were predicted using Smart (https://meme-suite.org/meme/) (Letunic et al., 2021) and visualized using TBtools. The 3D protein structure of BnaCYP86A4 (BnaA09G0695600ZS) in ‘4074’ and ‘4075’ was predicted using SWISS-MODEL (https://swissmodel.expasy.org) (Waterhouse et al., 2018).

## 3 Results

### 3.1 Phenotypic characteristics of ‘4074’ and ‘4075’

**Figure 1:**
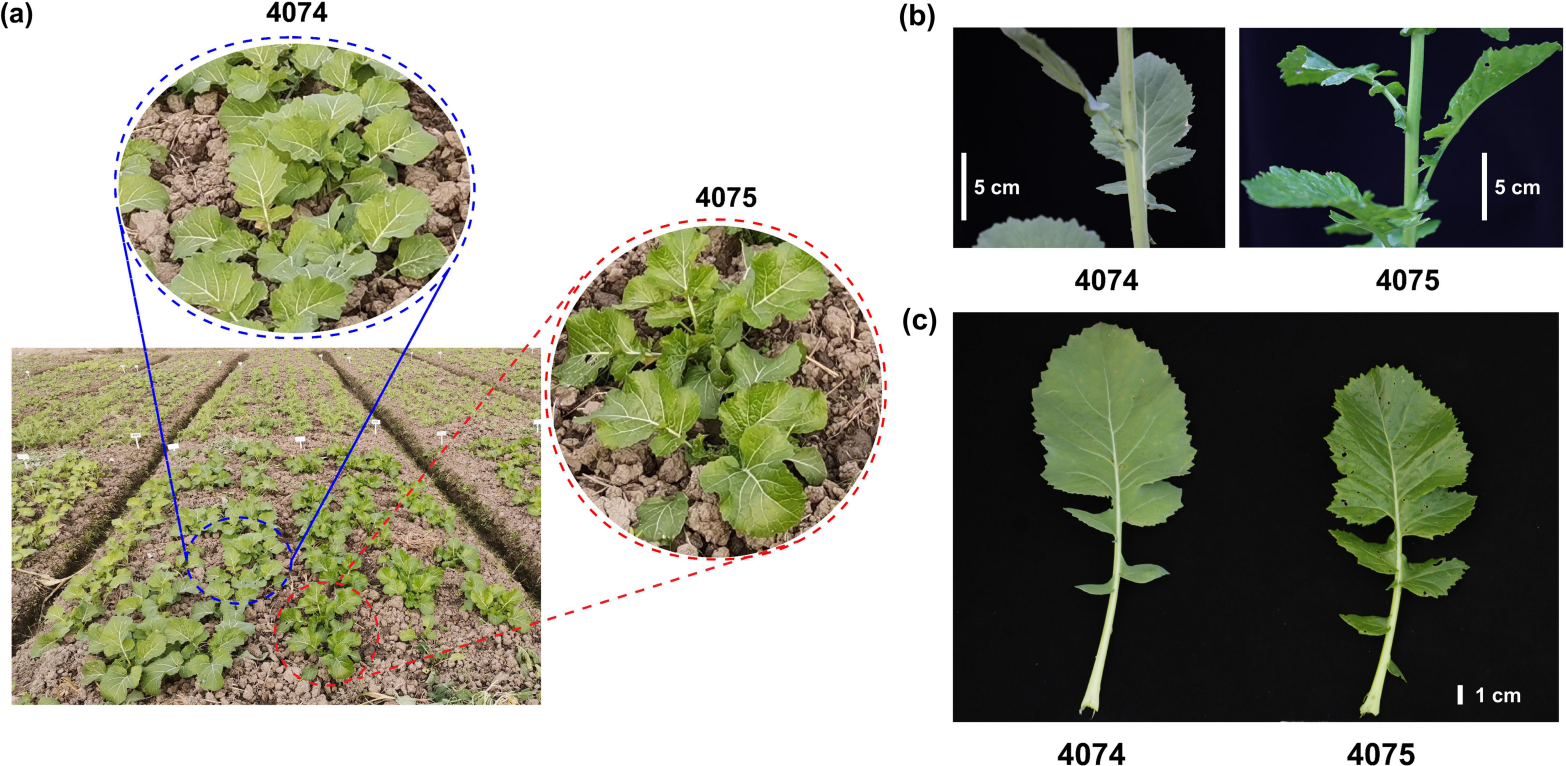
Comparison of cuticular wax coverage phenotype between the lines ‘4074’ and ‘4075’ by visual inspection. **(a)** Cuticular wax coverage phenotype on rapeseed seedlings in the field; **(b)** Cuticular wax coverage phenotype on the short petiole leaf in the mid-sect stem at the flower bud stage; and **(c)** Cuticular wax coverage phenotype on the mid-sect stem at the flower bud stage.

The *39J7H* mutant, characterized by its glossy phenotype, was identified in a large glaucous breeding population in the year 2008. To elucidate the nature of this mutation, we performed self-fertilization of the *39J7H* mutant utilizing a non-woven bag to aid self-pollination and avoid cross-pollination. Seeds obtained from the *39J7H* mutant were subsequently harvested for further experimentation. Surprisingly, the F_1_ population of mutant selfing progeny exhibited segregation of the waxy trait, with individuals displaying either the glossy mutant phenotype or glaucous wild-type phenotype. Phenotypic statistical analysis revealed a segregation ratio of 3:1 (173 glossy plants to 67 glaucous plants; $\chi^{2}=1.09<\chi_{0.05,1}^{2}=3.84$). Therefore, we concluded that the mutant trait is controlled by a single dominant nuclear gene. From the F_1_ segregating population, three glossy and three glaucous plants were selected for successive self-crossing to obtain the stable pure sister lines ‘4074’ (glaucous) and ‘4075’ (glossy) (**Figure 1a**).

Plants of the line ‘4074’ exhibited a glaucous phenotype starting from the fourth true leaf stage, whereas plants of line ‘4075’ presented a bright green cooler and a glossy phenotype on both the leaf and stem surfaces throughout the entire growth period (**Figure 1**). As near-isogenic lines (NILs) with similar genetic backgrounds but distinct wax phenotypic differences, ‘4074’ and ‘4075’ provide an advantageous model for the identification of genes that regulate cuticular wax synthesis. An F_1_ population resulting from the cross of ‘4074’ and ‘4075’, as well as an F_2_ population, were constructed for genetic analysis. The F_1_ plants of ‘4074’ × ‘4075’ exhibited a glossy phenotype, indicating that the glossy trait is dominant. In the F_2_ population, the phenotypic segregation ratio was 3:1 (147 glossy plants to 52 glaucous plants; $\chi^{2}=0.16<\chi_{0.05,1}^{2}=3.84$). These results suggest that a single Mendelian locus governs glossy traits.

### 3.2 Wax crystal morphology and chemical changes in leaf cuticular wax of ‘4075’

**Figure 2:**
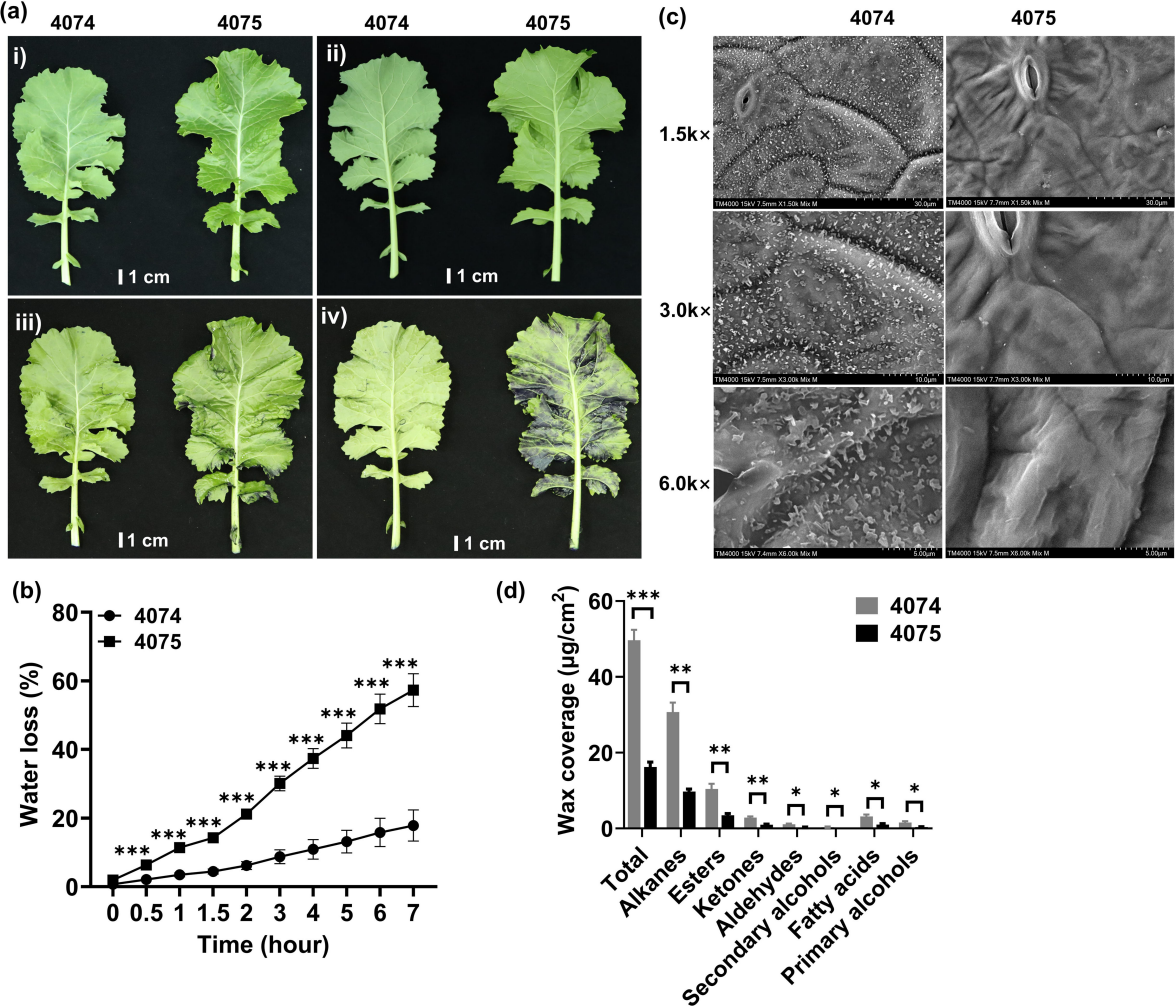
Epicuticular wax deposition on leaves of ‘4074’ and ‘4075’. **(a)** Toluidine blue staining pattern of ‘4074’ and ‘4075’. 4074 (left), 4075 (right). Before staining (a-i, the front of the leaf; a-ii, the back of the leaf), after staining (a-iii, the front of the leaf; a-iv, the back of the leaf). **(b)** Water loss rate of leaves of ‘4074’ and ‘4075’. **(c)** Epicuticular wax crystals on ‘4074’ and ‘4075’ leaf surfaces as detected by SEM at 1.5k ×, 3.0k ×, and 6.0k × magnification, respectively. **(d)** Cuticular wax composition in rapeseed ‘4074’ and ‘4075’ leaves.

Cuticular wax defects in plants often lead to increased leaf permeability (Li et al., 2026; Tao et al., 2024; Zhang et al., 2025). To investigate this, we incubated the fourth leaf from the growth point during the seven-leaf and one-heart stage of ‘4074’ and ‘4075’ plants in 0.05% (w/v) Toluidine Blue (TB) solution 10 minutes and assessed the staining intensity. Leaves of ‘4074’ showed barely stained whereas many areas of ‘4075’ leaves were heavily stained (**Figure 2a**). Furthermore, during 0.5–8 h after stomatal closure, ‘4075’ showed a significantly higher rate of water loss compared to ‘4074’ (**Figure 2b**). These results are consistent with the observation of an abnormal cuticular layer in the leaves, suggesting the glossy ‘4075’ plants were compromised in the strength of water permeability barrier.

To determine whether the deposition of epicuticular wax crystals on the leaf surface contributed to the glaucous phenotype and the increased water permeability, scanning electron microscopy (SEM) was used to assess the density of wax crystals on leaf tissue in both lines. SEM analysis revealed a significant reduction in wax crystals on certain regions of the leaf surface in ‘4075’ plants (**Figure 2c**). In contrast, ‘4074’ plants displayed abundant rod-shaped, sheet-like, and tubular wax crystals, contributing to the white waxy coating observed on their leaf surfaces (**Figure 2**). Additionally, the leaves of ‘4075’ plants exhibited a relatively smooth surface and a crumpled appearance. These findings suggest that the glossy phenotype of ‘4075’ results from a marked reduction in epicuticular wax crystals on the leaves (**Figure 2c**).

To evaluate variations in wax compounds responsible for the glossy phenotype, gas chromatography-mass spectrometry (GC-MS) was used for quantitative and compositional analysis of leaf wax extracts. GC-MS results revealed that the cuticular wax profiles of these lines were similar to those reported by Pu et al. (2013) and Tassone et al. (2016). Leaf wax consists primarily of alkanes, alkyl esters, ketones, fatty acids, aldehydes, primary alcohols, and secondary alcohols. A distinct difference in wax composition was observed between glaucous and glossy lineages (**Figure 2d**, **Supplementary Table 1**). The total wax coverage in ‘4075’ was 16.2 μg/cm^2^, representing a 67.3% reduction compared with the ‘4074’ (**Supplementary Table 1**). In ‘4075’, significant reductions were observed across all major wax components, including alkanes, alkyl esters, ketones, fatty acids, aldehydes, primary alcohols, and secondary alcohols, with alkanes, alkyl esters, ketones, and secondary alcohols showing the most pronounced decreases (**Supplementary Table 1**). Previous studies have shown that alkanes, ketones, and secondary alcohols are key contributors to total wax, and their deficiency negatively affects the overall wax deposition (Rashotte et al., 1997; Samuels et al., 2008). These results indicate that the glossy phenotype of ‘4075’ is primarily due to disruptions in cuticular wax formation.

### 3.3 BSA-seq technology identifies a QTL associated with cuticular wax on chromosome A09

**Figure 3:**
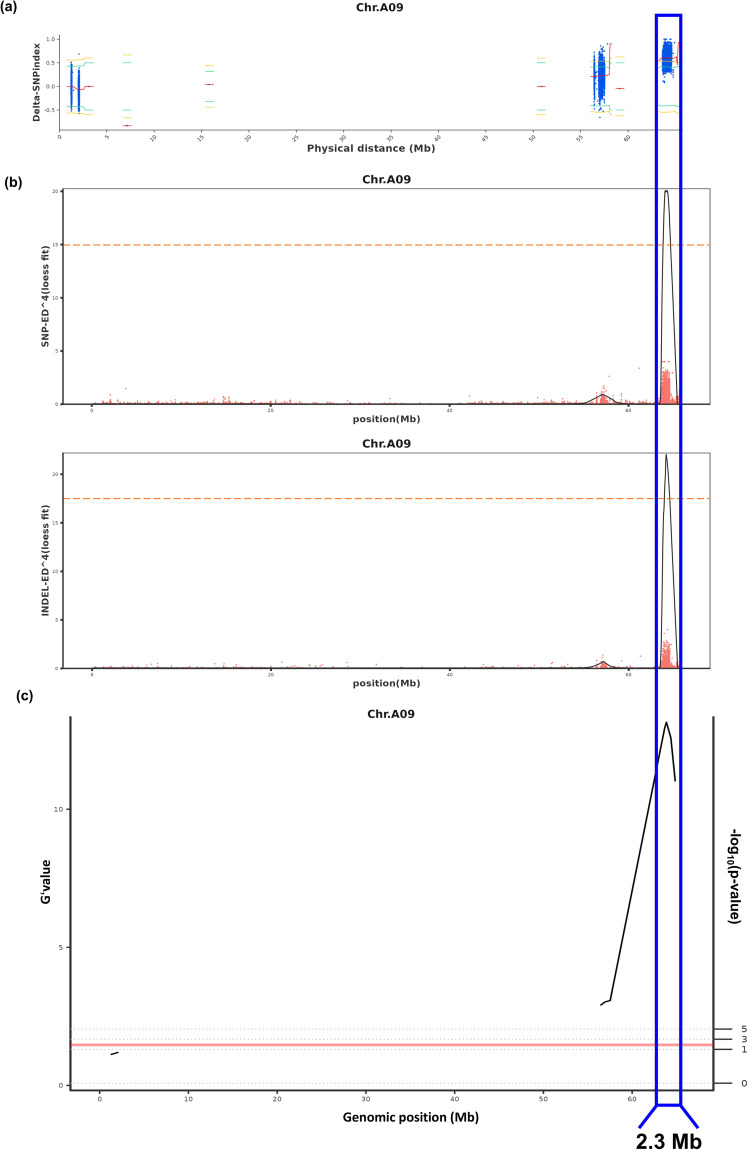
Quantitative trait locus (QTL) analysis of rapeseed leaf cuticular wax using 3 QTL-seq methods and position of the candidate interval of *qCWA9.1* on rapeseed chromosome A9. **(a)** Identification of SNP/Indels on Chr.A09 associated with cuticular wax using Δ(SNP-index) method. Green indicates the line of 95% confidence, yellow indicates the line of 99% confidence, and red indicates the line of the mean value of Δ (SNP-index). **(b)** Identification of SNP/InDels on Chr.A09 associated with cuticular wax using Euclidean distance (ED) method. **(c)** Identification of SNP/Indels on Chr.A09 associated with cuticular wax using G’value method. The blue highlights the significant genomic regions of 62.70–65.0 Mb on Chr.A09.

Phenotyping analysis of cuticular wax from the F_2_ mapping population was conducted, and libraries were constructed using two extreme bulks and two parental bulks. Whole-genome sequencing was performed using the Illumina NovaSeq system, yielding high-quality sequencing data, with an average Q20 of 98.2% and a Q30 of 94.9% (**Supplementary Table 2**). Δ(SNP-Index) values (**Supplementary Figure 2a**), Euclidean distance (ED) values (**Supplementary Figure 2b**), and G-values (**Supplementary Figure 2c**) were used to identify candidate leaf cuticular wax QTL regions in rapeseed. The SNP index for each identified SNP was calculated using a 1 kb sliding window at 1 Mb intervals and plotted for the glossy bulk and glaucous bulk (**Supplementary Figure 2a**). The SNP-index data from both bulks were combined to calculate and plot Δ (SNP-index) at genomic locations (**Supplementary Figure 2a**). At the 99% statistical confidence level, a 2.3 Mb interval from 62.70 to 65.0 Mb on chromosome A09 was observed to be significantly associated with cuticular wax synthesis and was designated as *qCWA9.1* (**Figure 3**, **Supplementary Figure S2a**). The region on chromosome A09 from 62.70 to 65.0 Mb displayed a higher average than 0.5 Δ (SNP-index) (**Figure 3b**). Interestingly, the major QTL identified by the ED method and the G’ value method was more significant than that identified by the SNP index method (**Supplementary Figures 2b, c**). It exhibited a distinct peak on Chr.A09 and was consistent with the SNP index results (**Figure 3**), indicating the accuracy and reliability of this QTL. The results suggest that a candidate gene controlling leaf cuticular wax biosynthesis is located within this 2.3 Mb region (**Supplementary Figure 3**).

Based on the reference genome and annotation information of the *Brassica napus* cultivar ‘ZS11’, 136 open reading frames (ORFs) were predicted within the 2.3 Mb region of *qCWA9.1* (**Supplementary Table 3**). Among these 136 candidate genes, 108 and 28 genes occurred in the nonsynonymous and synonymous SNV, respectively (**Supplementary Table 3**). The mapping regions identified in this study, along with the previously reported mapped intervals of the dominant glossy mutant of *BnaA.GL* (Pu et al., 2013) were both located on chromosome A9. *BnaCER1* (*BnaA09G0721400ZS*), previously identified as the candidate gene involved in cuticular wax synthesis (Pu et al., 2013), was located at nucleotides 65832114–65836706 at the bottom of the mapping region in this study. We cloned the genomic sequence of *BnaCER1* in the parents ‘4074’ and ‘4075’ and aligned with the *BnaCER1* sequence from the glossy mutant *BnaA.GL*, which suggested no differences in the *BnaCER1* sequence between ‘4074’ and ‘4075’ (**Supplementary Figure 3**). Interestingly, the *BnaCER1* sequence in ‘4074’ and ‘4075’ was identical to that of the WT in the previous study (Pu et al., 2013). This finding inspired us and raised the possibility that a novel gene might be involved in cuticular wax synthesis.

### 3.4 Fine mapping of *qCWA9.1*

**Figure 4:**
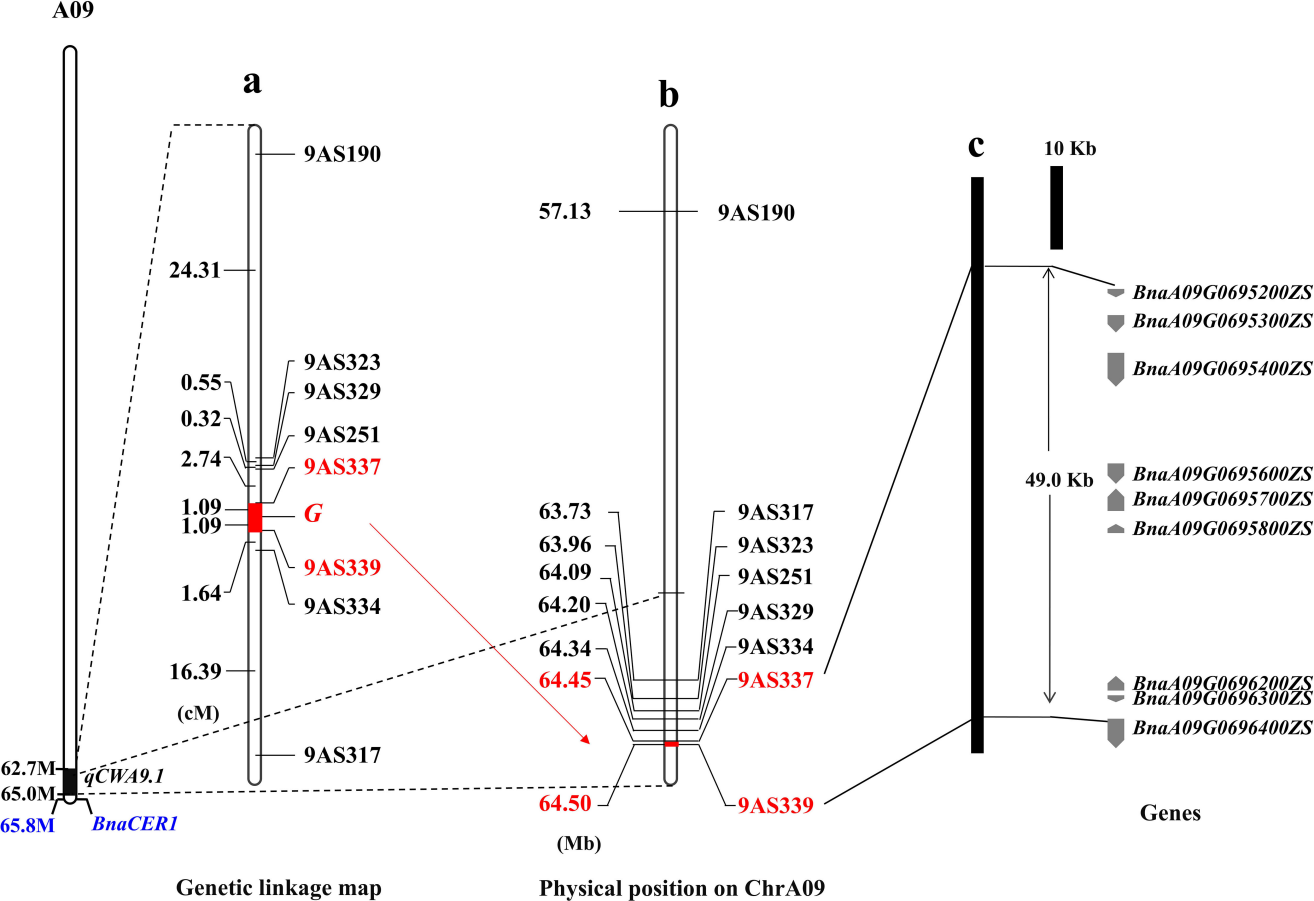
Fine-scale map of *qCWA9.1* for cuticular wax synthesis on *Brassica napus* ChrA09 with candidate genes. **(a)** Genetic linkage map of the *qCWA9.1* region. *qCWA9.1* was mapped to the region between markers 9AS337 and 9AS339 using 199 F_2_ individuals and combined with phenotyping of the F_2:3_ population. The numbers on the left side of the chromosome indicate the genetic distance (cM) between the adjacent markers. **(b)** Fine mapping interval for *qCWA9.1* locus and its corresponding physical map of chromosome A09 on rapeseed. The numbers on the left side of the chromosome indicate the physical position of markers. **(c)** Candidate region of *qCWA9.1* and the annotated genes in the *Brassica napus* multi-omics information resource database (https://yanglab.hzau.edu.cn/BnIR) (Yang et al., 2023). The mapping region of *qCWA9.1* was narrowed down to a 49.0-kb interval of the genome that included nine candidate genes. Arrows indicate the length of genes and direction of genes arrangement on the chromosome. Detailed information on these nine genes is provided in **Table 1**.

To narrow down the candidate region of *qCWA9.1*, SSR markers were developed within a 2.3-Mb interval for further fine mapping *qCWA9.1*. In total, 162 primer pairs were used to screen the two parental lines, resulting in the identification of 11 markers that amplified polymorphisms between ‘4074’ and ‘4075’. These 11 polymorphic markers were screened in an F_2_ population consisting of 147 individuals with a glossy phenotype and 52 with a glaucous phenotype. Among these, eight primer pairs exhibited polymorphisms and stable, scorable band patterns across all F_2_ individuals and were retained for genetic mapping. The glossy and glaucous phenotypes were accurately distinguished by markers 9AS337 and 9AS339. This result indicated that markers 9AS337 and 9AS339 were tightly linked to the cuticular wax. Moreover, a morphological marker, denoted as G, was obtained from the leaf phenotype of the F_2:3_ population. Eight polymorphic markers (9AS190, 9AS323, 9AS329, 9AS251, 9AS337, 9AS339, 9AS317, and 9AS334) with stable, and clear spectra and one morphological marker (G) were selected for linkage map construction (**Figure 4a**). *qCWA9.1* was ultimately delimited to a 49-kb genomic region between markers 9AS337 and 9AS339 (**Figure 4**). Examination of the ‘ZS11’ reference genome revealed nine predicted genes within this 49-kb region (**Table 1**, **Figure 4b**).

**Table 1:** Prediction of candidate genes within the 49-Kb gene-mapped region on chromosome A09.

Gene ID	Location	Gene annotations
*BnaA09G0695200ZS*	64453189-64454162	Erythronate-4-phosphate dehydrogenase family protein
*BnaA09G0695300ZS*	64456041-64458047	Probable serine/threonine-protein kinase
*BnaA09G0695400ZS*	64460591-64464482	Ferric reduction oxidase 2, FRO2; Encodes the low-iron-inducible ferric chelate reductase responsible for reduction of iron at the root surface
*BnaA09G0695600ZS*	64473392-64475747	Cytochrome P450, CYP86A4; Encodes a member of the CYP86A subfamily of cytochrome p450 proteins; Fatty acid omega-hydroxylase
*BnaA09G0695700ZS*	64476239-64478855	Glycerol-3-phosphate 2-O-acyltransferase 4, GPAT4; bifunctional sn-glycerol-3-phosphate 2-O- acyltransferase/phosphatase. Involved in cutin assembly and is functionally redundant with GPAT8
*BnaA09G0695800ZS*	64480339-64481411	Aquaporin PIP1-3, PIP1-3; localizes to the plasma membrane and exhibits water transport activity in Xenopus oocyte
*BnaA09G0696200ZS*	64498002-64499704	Phosphatidylinositol N-acetyglucosaminlytransferase subunit P-related
*BnaA09G0696300ZS*	64500167-64500967	Unknown protein
*BnaA09G0696400ZS*	64502753-64506138	Phosphatidylcholine-retinol O-acyltransferase

### 3.5 Sequence analysis and expression pattern of the candidate genes in *qCWA9.1*

According to the gene annotation information of the *Brassica napus* resources database (https://yanglab.hzau.edu.cn/BnIR) (Yang et al., 2023), eight annotated or predicted genes and one functionally unknown gene were located in the target DNA sequences of the 49.0-kb region (**Figure 4b**, **Table 1**). Among the nine predicted genes, *BnaA09G0695600ZS* and *BnaA09G0695700ZS* garnered significant attention. These two genes were predicted to encode a member of the CYP86A subfamily of cytochrome p450 proteins (CYP86A4) and glycerol-3-phosphate 2-O-acyltransferase 4 (GPAT4). Both genes were prioritized for further analysis due to their known involvement in cutin and wax biosynthesis (Li-Beisson et al., 2009; Rupasinghe et al., 2007; Zhang et al., 2024).

First, we performed sequence analysis of the nine candidate genes in the coding regions between the ‘4074’ and ‘4075’. Compared with ‘4074’, all nine genes in ‘4075’ were found to have multiple Indel and SNP variants in their exons (**Supplementary Table 3**). The variation types included indel insertions, deletions, single-base insertions, and substitutions (**Supplementary Table 3**). Analysis of the amino acid Sequence predictions of the proteins encoded by these gene showed that the mutations in CDS sequence were non-synonymous mutations in *BnaA09G0695200ZS*, *BnaA09G0695300ZS*, *BnaA09G0695400ZS*, *BnaA09G0695600ZS*, *BnaA09G0695800ZS*, *BnaA09G0696200ZS*, *BnaA09G0696300ZS*, and *BnaA09G0696400ZS*, whereas *BnaA09G0695700ZS* occurred as synonymous mutations in ‘4075’ (**Supplementary Table 4**, **Supplementary Figure 4**) sequences and conserved domains revealed that BnaA09G0695800ZS was terminated prematurely in ‘4075’, lacking its entire conserved structural domain. Five amino acid sequences, BnaA09G0695300ZS, BnaA09G0695400ZS, BnaA09G0695600ZS, BnaA09G0696200ZS, and BnaA09G0696400ZS, in ‘4075’ exhibited 1–3 amino acid substitutions in their conserved domains. The amino acid sequence of the functionally unknown gene *BnaA09G0696300ZS* did not predict any conserved domains. Although BnaA09G0695200ZS exhibited amino acid substitutions, deletions, and insertions, the conserved domain remained unchanged. *BnaA09G0695700ZS*, a gene of particular interest, showed no changes in either its amino acid sequence or conserved domains.

**Figure 5:**
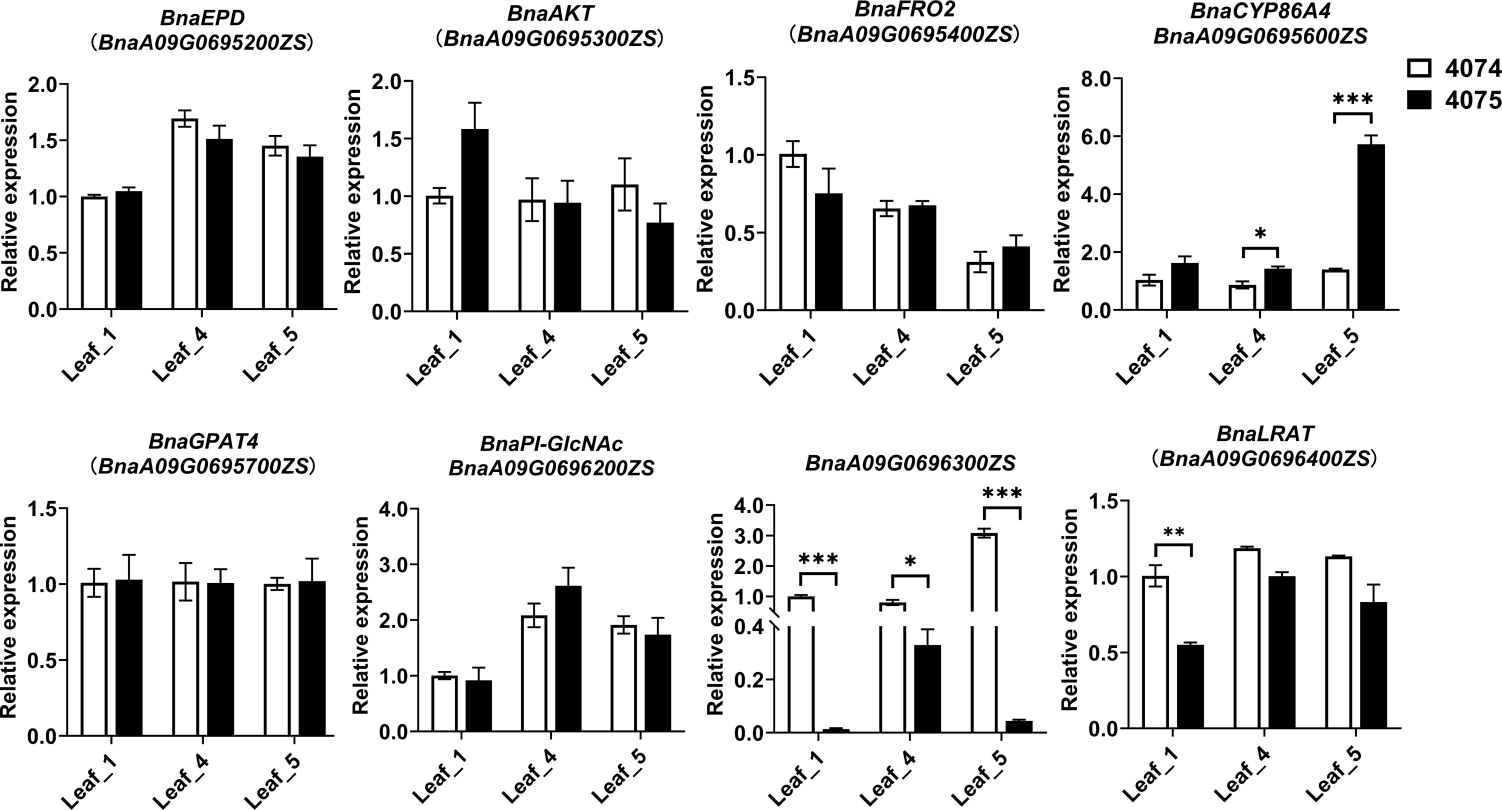
Expression patterns of candidate genes correlated with cuticular wax synthesis in Brassica napus between ‘4074’ and ‘4075’. Data are presented as the means of three biological replicates (mean ± SD). Samples were collected at the stages of the first (15 d), fourth (45 d), and fifth (62 d) true leaves of the rosette after sowing seeds in a substrate grown in a greenhouse. The plants of ‘4074’ began to exhibit the waxy phenotype at the fourth true leaf stage, while ‘4075’ plants consistently displayed glossy leaves. Asterisks indicate significant differences between ‘4074’ and ‘4075’ at the same stage (*p < 0.05; **p < 0.01; ***p < 0.001, Student’s t-test).

To determine whether mutations in the cDNAs of these genes were responsible for the glossy phenotype, the expression patterns of the nine genes, in ‘4075’ relative to ‘4074’ were analyzed by qRT-PCR at growth stages of the first true leaf (Leaf_1), fourth true leaf (Leaf_4), and fifth true leaf (Leaf_5). Among the nine genes, the transcript of *BnaA09G695800ZS* was hard to detect from the leaves at all stages examined even at 35 cycles of amplification. Differential expression analysis revealed significant changes in two of the remaining eight genes between ‘4074’ and ‘4075’. *BnaA09G696300ZS* was significantly downregulated at all investigated stages, while *BnaA09G695600ZS* was significantly upregulated during waxy formation (Leaf_4 and Leaf_5) in ‘4075’ compared with ‘4074’. *BnaA09G695600ZS* was significantly upregulated by 4.32-fold at the fifth-leaf stage in the glossy line ‘4075’ (**Figure 5**).

In summary, the two genes showed differences in both amino acid sequence and expression patterns between ‘4074’ and ‘4075’ (**Supplementary Table 5**). Among these, one functional gene, *BnaA09G695600ZS*, was considered a putative candidate based on homologous gene functions in *Arabidopsis thaliana* (**Table 1**). *BnaA09G695600ZS* is a homologue of *AT1G01600* (*AtCYP86A4*), a fatty acid omega-hydroxylase and a member of the CYP86 subfamily of P450 enzymes, which is involved in cutin synthesis according to previous studies (Li-Beisson et al., 2009; Rupasinghe et al., 2007). However, another copy of *CYP86A4*, *BnaA10g00380D*, in *Brassica napus* has been reported to be a candidate gene controlling leaf cuticular wax biosynthesis (Jin et al., 2020; Long et al., 2023). This is similar to the phenotypic and biochemical changes observed in this study. Therefore, we speculate that *BnaA09G695600ZS* serves as one of candidate genes for regulating cuticular wax formation in this study.

### 3.6 Sequence analysis of *BnaCYP86A4*

**Figure 6:**
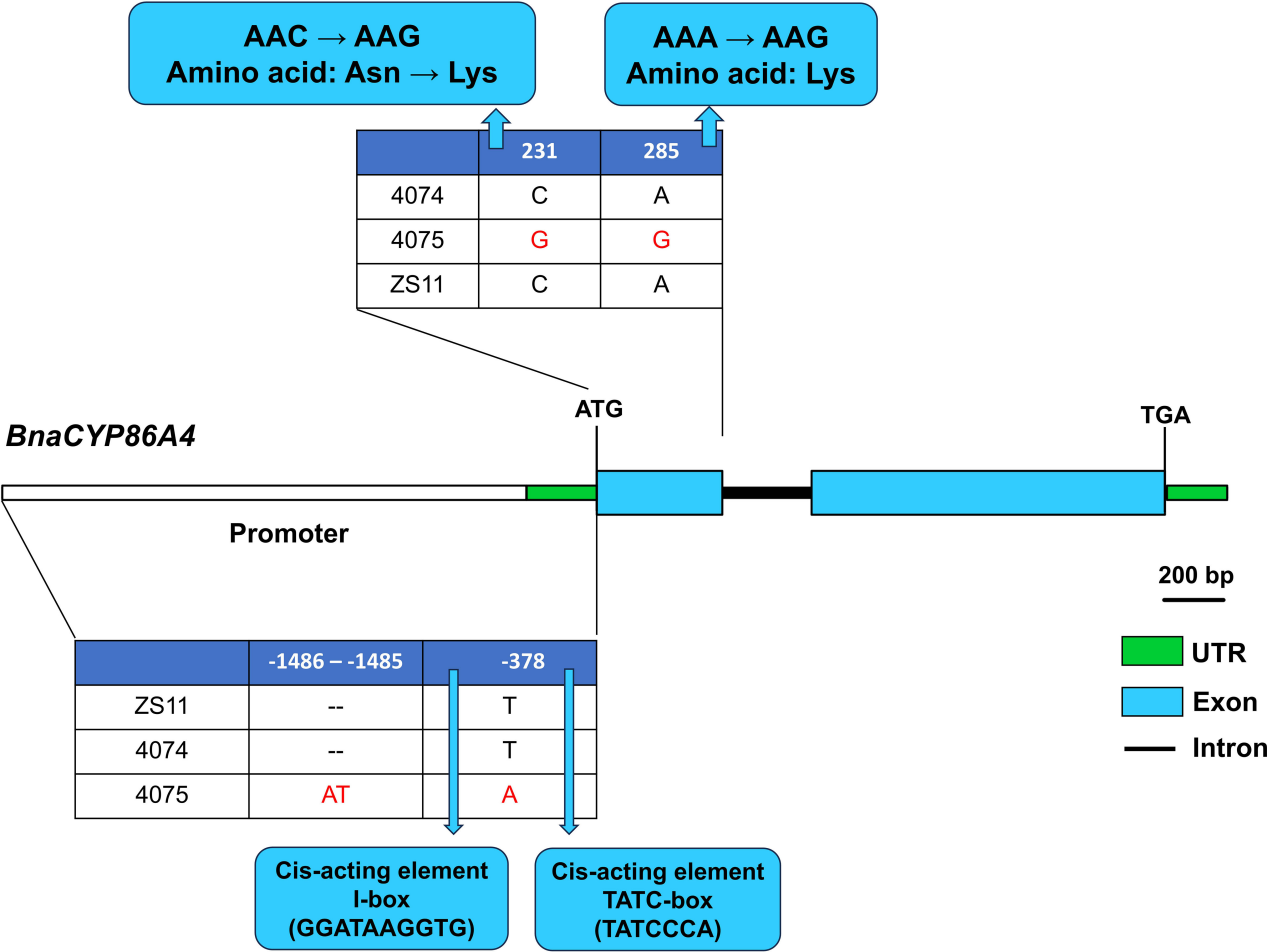
Gene structure and nucleic acid variation in the *BnaA09G0695600ZS* among ‘4074’, ‘4075’, and ‘ZS11’. SNPs variations are indicated by red symbols and shown at their relative positions. Amino acid changes and functional *cis*-element prediction of the promoter at the mutated locus are indicated by the blue boxes.

Genomic DNA (gDNA), coding sequences (CDS), and the 2-kb promoter region of *BnaCYP86A4* from both parents ‘4074’ and ‘4075’ were cloned by RT-PCR. No nucleotide variations were detected in the gDNA or CDS sequences of *BnaCYP86A4* in ‘4074’ and the reference genome ‘ZS11’. In contrast, three nucleotide variations were identified in the gDNA, two SNPs in the CDS, and three nucleotide variations in the promoter sequences of *BnaCYP86A4* in the parent ‘4075’ (**Figure 6**). Compared with ‘4074’, a C to G transition at nucleotide 231 in exon1 caused a Lys to Asn substitution at the 77th amino acid, while an A to G transition at nucleotide 285 in exon1 did not result in any amino acid change in *BnaCYP86A4* in ‘4075’. Additionally, a T to C transition at nucleotide 640 in the intron was observed in the gDNA of *BnaCYP86A4*.

Consistent with the differences in expression levels, three single-nucleotide polymorphisms (SNPs) were identified in the 2 kb promoter region of *BnaA09G695600ZS* between ‘4074’ and ‘4075’, including a T to A transition at nucleotide -378 and an AT insertion between nucleotides -1486 and -1485 in ‘4075’ (**Figure 6**). Notably, the SNP variation at position -378 was located within the *cis*-acting elements, the TATC-box and the I-box, resulting in the absence of both the TATC-box and the I-box in the *BnaCYP86A4* promoter in ‘4075’. Interestingly, the TATC-box and I-box are commonly found as cis-elements in plant inducible promoters (Luo et al., 2013; Nardia et al., 2014; Zhang et al., 2014). The TATC-box is associated with responsiveness to gibberellin (GA), abscisic acid (ABA), and ethylene (ETH), whereas the I-box responds to light, GA, and ABA. Additionally, a member of the CYP86 clan, *OsCYP96B4*, has been reported to influence lipid metabolism by fine-tuning the GA-to-ABA balance (Tamiru et al., 2015; Wang et al., 2016). Additionally, overexpression of CYP714D1 in transgenic poplar enhanced growth and salt tolerance by regulating gibberellin and ion homeostasis (Gao et al., 2021). Moreover, multiple phytohormone-responsive *cis*-elements were predicted in the promoter region of *BnaCYP86A4* (**Supplementary Figure 5**). Therefore, it can be hypothesized that the absence of the TATC-box in the *BnaCYP86A4* promoter in ‘4075’ may disrupt lipid metabolism by altering GA and ABA responsiveness, potentially contributing to the glossy phenotype.

**Figure 7:**
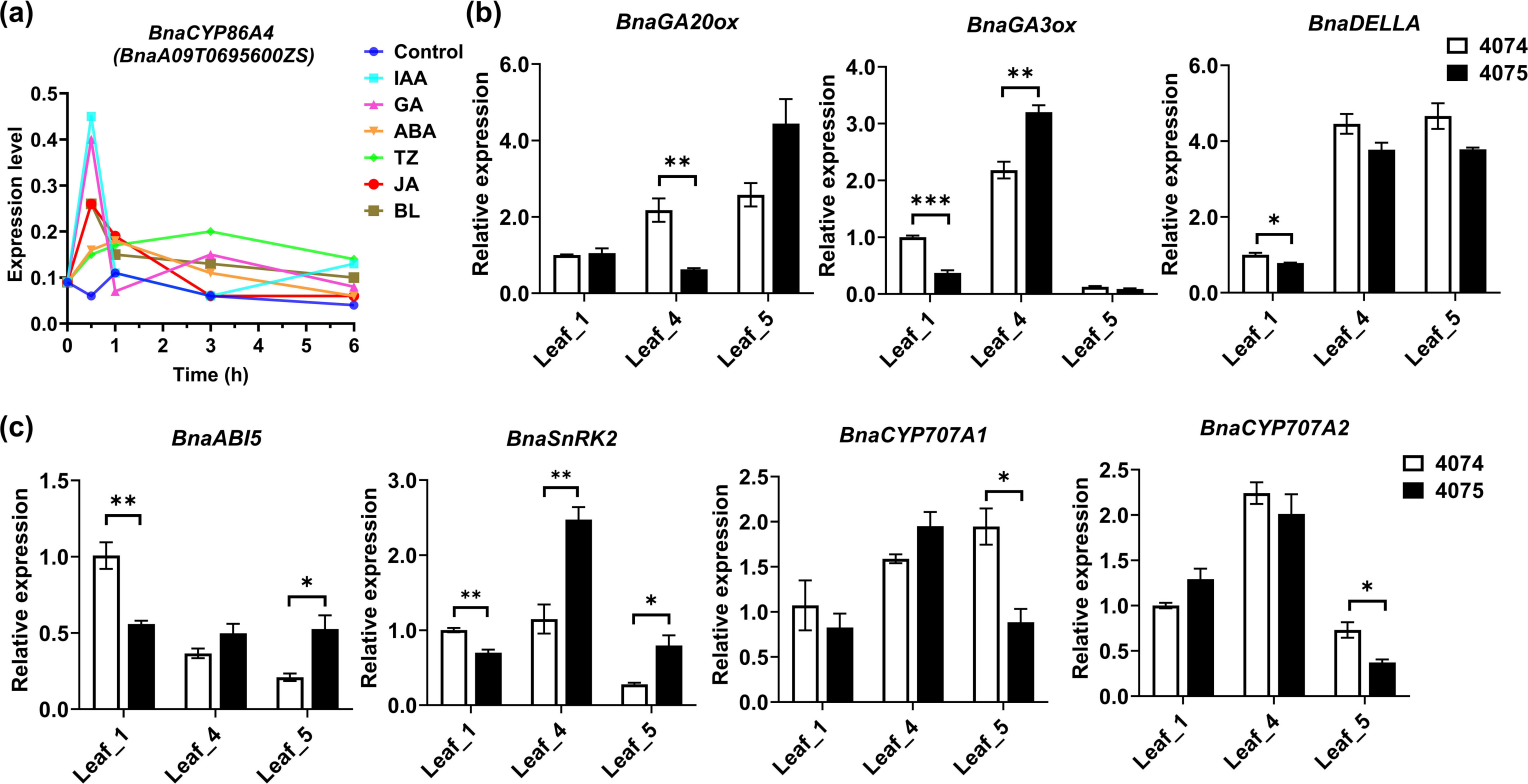
Expression pattern of *BnaCYP86A4* treated with multiple hormones and expression profile of responsive genes in GA and ABA signaling and metabolism pathways. **(a)** Expression pattern of *BnaCYP86A4* treated with multiple hormones in rapeseed leaf. Expression data were obtained from the BnIR database (https://yanglab.hzau.edu.cn/BnIR). **(b)** Expression profile of genes in GA signaling and metabolism pathway between ‘4074’ and ‘4075’. **(c)** Expression profile of genes in ABA signaling and metabolism pathway between ‘4074’ and ‘4075’. IAA, Indole-3-acetic acid; GA, Gibberellin; ABA, Abscisic Acid; TZ, Zeatin; JA, Jasmonic acid; BL, Brassinolide; GA20ox, GA 20-oxidase; GA3ox, GA 3-oxidase. Asterisks indicate significant differences between ‘4074’ and ‘4075’ at the same stage (*, p < 0.05; **, p < 0.01; Student’s t-test).

To confirm this hypothesis, we performed an expression profile analysis of genes involved in ABA and GA signaling and metabolism pathways. The expression of *BnaCYP86A4* in transcriptional level induced by various hormones (IAA, GA, ABA, TZ, JA, and BL) in leaves of ZS11were retrieved from the BnIR database (**Figure 7a**). After 0.5 hours of treatment with IAA, GA, JA, BL, ABA, and TZ, the expression of *BnaCYP86A4* was sharply upregulated (**Figure 7a**), indicating that the *BnaCYP86A4* responds to multiple hormones in leaves. This was consistent with the presence of multiple hormone-responsive elements in the promoter region of *BnaCYP86A4* (**Supplementary Figure 5**). Notably, ABA and TZ have lasting inducing effects on the expression of *BnaCYP86A4.* In addition, the GA biosynthesis genes *BnaGA20ox* and *BnaGA3ox* exhibited significant downregulation and upregulation at the leaf_4 stage, respectively. GA20oxs is responsible for the production of C20-GAs using C19-GAs as substrates, while GA3ox enzymes hydroxylate the inactive precursors GA9 and GA20 to form the biologically active hormones GA_4_ and GA_1_ (Gao et al., 2017). The significant downregulation of GA20ox will lead to a reduction in GA_19_, the precursor of bioactive GA_1_, in ‘4075’. However, the significant increase in GA3ox expression in leaves may promote the production of bioactive GA_4_, resulting in no change in the overall levels of bioactive GA due to the lack of significant changes in the expression of the GA signaling response factor DELLA in ‘4075’. Surprisingly, the ABA signal response genes *ABI5* and *SnRK2* showed significant upregulation in ‘4075’, and the ABA catabolic gene *CYP707A* exhibited significant downregulation at the leaf_5 stage, indicating ABA accumulation in the leaves of ‘4075’. Therefore, we hypothesis that the accumulation of ABA in ‘4075’ might disrupt the balance between ABA and GA, promoting the expression of *BnaCYP86A4* and disrupting the lipid metabolism. Based on these results, we speculate that *BnaA09G695600ZS* is one of candidate gene responsible for the wax phenotype.

### 3.7 Analysis of phylogenetic tree and homology sequence of BnaCYP86A4

Each plant species contains a substantial number of P450 enzymes that play diverse roles in biosynthetic and catabolic pathways (Hansen et al., 2021). In allotetraploid species such as *Brassica napus*, a greater number of P450 copies have been identified, with 765 P450s reported within its genome. However, the functions of many of these proteins remain poorly understood (Xue et al., 2024). To place BnaA09G0695600ZS within the P450 evolutionary tree and gain insights into its potential functions, the full-length amino acid of BnaA09G0695600ZS was employed as a query to identify its closest relatives in the proteomes of *Brassica napus*, *Arabidopsis*, rice, and wheat. A total of 145 putative protein sequences related to BnaCYP86A4 have been identified in *Brassica napus* (43), *Arabidopsis* (21), rice (16), and wheat (65).

**Figure 8:**
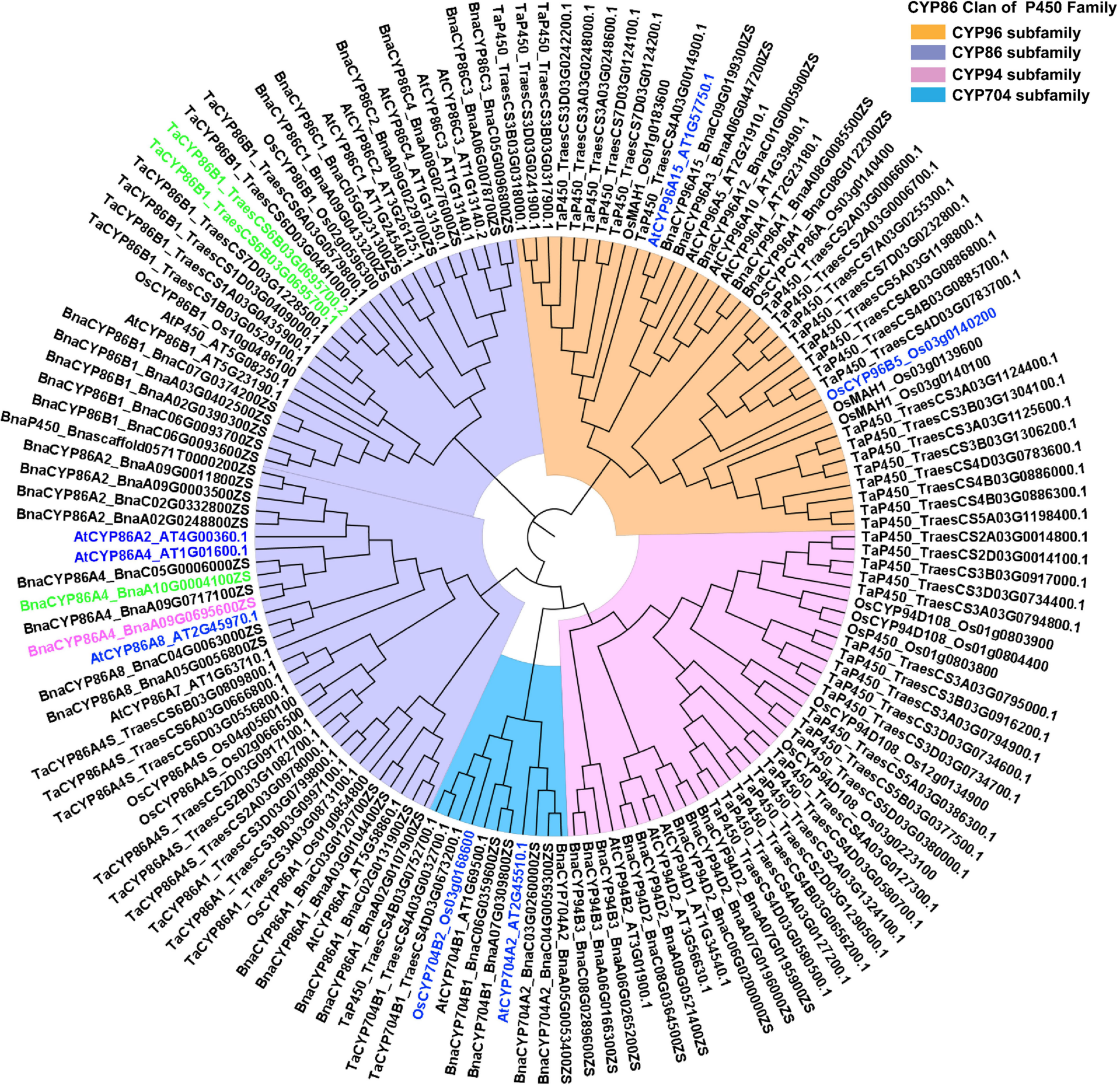
Protein phylogeny of BnaCYP86 and its’ related P450 proteins. A maximum likelihood analysis was conducted using MEGA 7.0, based on the alignment of BnaCYP86A4 with the most similar P450 sequences from *Brassica napus* (Bna), Arabidopsis (At), rice (Os), and *Triticum aestivum* (Ta). Bootstrap values represent the percentage of 1,000 replicates. The blue font indicates CYP86 subfamily members known to participate in plant cuticle synthesis, the rose font indicates the position of the target gene (BnaCYP86A4_BnaA09G0695600ZS), and the green font indicates the members of the CYP86 subfamily as candidate genes to regulate the wax formation by GWAS and RNA-seq, liposomes in *Brassica napus* and wheat (Jin et al., 2020; Long et al., 2023; Wen et al., 2021). The proteins closest to BnaCYP86A4 belonged to four families (CYP86, CYP96, CYP94, and CYP704) all within the CYP86 clan of P450 monooxygenases, which are indicated in orange, purple, pink, and blue blocks, respectively.

Phylogenetic analysis indicated that 145 putative protein sequences related to BnaCYP86A4 all belong to four distinct families, CYP86, CYP96, CYP94, and CYP704, were situated in distinct branches, which collectively formed the CYP86 clan of P450 monooxygenases (**Figure 8**). This result suggests that BnaCYP86A4 belongs to the CYP86 clan of P450 monooxygenases. CYP86 enzymes have been found that generally use fatty acids, fatty alcohols, alkanes, and their derivatives as substrates (Liu et al., 2025; Pinot and Beisson, 2011). Phylogenetic analysis showed that BnaCYP86A4 was closely related to its putative counterparts in *Arabidopsis* (AtCYP86A4, AtCYP86A2, and AtCYP86A8), rice (Os02g0666500 and Os04g0560100), and wheat (TraeSC2D03G0901700.1, TraeSC2B03G1082700.1, and TraeSC2A03G0978000.1) (**Figure 8**). However, at present, only CYP86A4 in *Arabidopsis* has been reported to catalyze the hydroxylation of fatty acids and to be involved in the synthesis of fatty acid-derived monomers for two major plant lipid polymers, cutin and suberin (Kollatukudy, 1981; Li-Beisson et al., 2009; Rupasinghe et al., 2007). There is no further information on the function of these CYP86A4 homologs in other species. In *Brassica napus*, four copies of *CYP86A4*, *BnaCYP86A4_A09a* (*BnaA09G0695600ZS*), *BnaCYP86A4_A09b* (*BnaA09G0717100ZS*), *BnaCYP86A4_A10* (*BnaA10G0004100ZS*), *BnaCYP86A4_C05* (*BnaC05G0006000ZS*) were identified in lines ‘4074’, ‘4075’, and the reference genome ‘ZS11’ (**Figure 8**). Among these, only *BnaA09G0695600ZS* exhibited sequence and expression differences between ‘4074’ and ‘4075’ in this study, while the other copies showed no differences in sequence or expression (**Supplementary Figures 6**-**S12**). Although CYP86A4 was initially characterized as a key enzyme for catalyzing cutin synthesis in *Arabidopsis*, it was identified here as a candidate gene associated with leaf cuticular wax in rapeseed. Previous studies also reported that another copy of *BnaCYP86A4*, *BnaA10g00380D*/*BnaA10G0004100ZS*, was identified as a candidate gene controlling leaf cuticular wax formation through GWAS and RNA-seq analyses (Jin et al., 2020; Long et al., 2023). Therefore, it is speculated that *CYP86A4* may play a role in cuticular wax synthesis.

**Figure 9:**
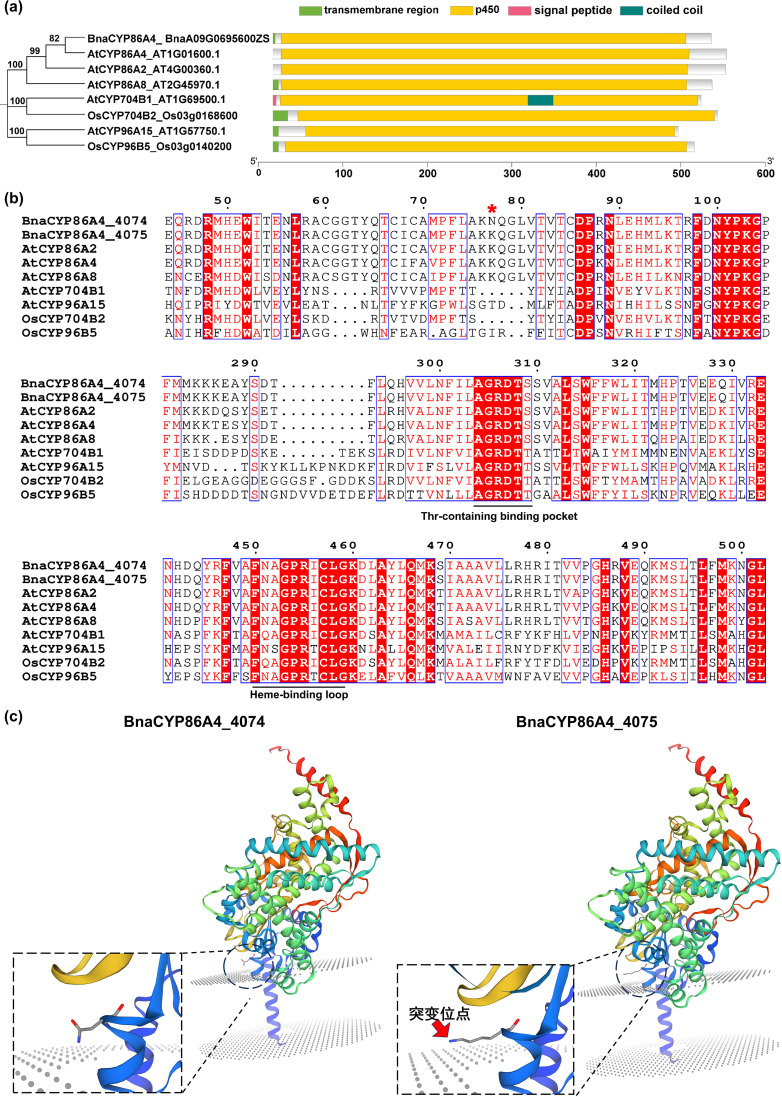
Sequence alignment of BnaCYP86A4 with several reported members of the CYP86A subfamily. **(a)** The conserved P450 domain, transmembrane region, signal peptide, and coiled coil were predicted in BnaCYP86A4 and several other members of the CYP86 subfamily. **(b)** The N77K mutation (marked by an asterisk) occurred within the P450 domain but did not affect the highly conserved catalytic center, including the heme-binding loop or Thr-containing binding pocket. **(c)** Three-dimensional protein structure prediction and mutation site identification of BnaCYP86A4_A09a in ‘4074’ and ‘4075’.

BnaCYP86A4_A09a is predicted to encode a cytochrome P450 protein consisting of 536 amino acids, featuring a common P450 domain (28–498 aa) and an 18-amino-acid transmembrane segment (ISNVMLLVAIVAAYWLWF) in the N-terminal region (**Figure 9a**). The amino acid sequences of BnaCYP86A shared 89.91%, 87.73%, 82.67%, 74.91%, 36.40%, 31.44%, 36.08%, and 35.34% similarity with BnaCYP86A4 (BnaA10g00380D/BnaA10G0004100ZS), AtCYP86A4, AtCYP86A2, AtCYP86A8, AtCYP704B1, AtCYP96A15, OsCYP704B2, and OsCYP96B5, respectively. Its high similarity to AtCYP86A4, AtCYP86A2 andAtCYP86A8 suggested that BnaCYP86A4 may perform a similar function, potentially playing a role in cutin biosynthesis. However, *BnaCYP86A4*, as a candidate gene implicated in the regulation of leaf cuticular wax, may also play a role in cuticular wax synthesis.

The primary structure of P450 proteins contains several motifs that are essential for their functional activity (Bak et al., 2011; Morant et al., 2003). These proteins include a heme-binding domain (FQAGPRICLG) incorporating the heme-binding consensus sequence FXXGXRXCXG at the C-terminus and a Thr-containing binding pocket (AGRDTT) for molecular oxygen, which is crucial for catalysis. In the *BnaCYP86A4* gene of the ‘4075’ line, a mutation (231 C to G) resulted in an amino acid substitution (Asn_77_Lys; **Figure 9b**, asterisk). This substitution occurred within the conserved p450 domain, however, outside the Thr-containing binding pocket and heme-binding loop, the catalytic center was conserved across all P450s (**Figure 9b**). Interestingly, the N_77_K mutation in BnaCYP86A4 of ‘4075’ mirrors a similar mutation observed in *Arabidopsis thaliana* CYP86A2 and CYP86A8, leading us to hypothesize that this mutation may not cause functional alterations (**Figure 9b**). However, three-dimensional (3D) protein structure prediction indicated that a change at the mutation site in BnaCYP86A4 of ‘4075’ (**Figure 9c**). Further genetic compensation experiments are required to validate whether this mutation affects protein function.

## 4 Discussion

### 4.1 *BnaCYP86A4_A9a* as one of candidate genes associated with leaf cuticular wax biosynthesis

In 2013, a novel dominant glossy mutant (*BnaA.GL*) of *Brassica napus* was characterized and genetically mapped (Pu et al., 2013). The *BnaA.GL* gene was localized to the end of chromosome A9 using a single-nucleotide polymorphism (SNP) chip assay combined with bulk segregant analysis (BSA). This study identified polymorphic markers on one side of the *BnaA.GL* gene but failed to detect markers on the other side of the gene. The marker *Bra032670* at the end of the chromosome is homologous to *AtCER1*. Therefore, *BnaCER1* (*BnaA09G0721400ZS*) was identified as one of the most downregulated genes in the *glossy* mutant and was proposed as a candidate gene. *BnaCER1* (*BnaA09G0721400ZS*) is located at positions 65832114–65836706 on ChrA09, which is downstream of the mapping region identified in the current study. Sequence analysis in a previous study revealed no significant sequence alterations between the wild-type (WT) and *GL* mutant of *BnaCER1*, except for three SNPs in the fifth intron. In this study, we cloned full-length *BnaCER1* and founded no differences in the sequence between ‘4074’ and ‘4075’. The sequences of *BnaCER1* in ‘4074’ and ‘4075’ were identical to the WT in the previous study (Pu et al., 2013) (**Supplementary Figure 3**). In this study, we also identified a dominant *glossy* mutant *39J7H* in the field, and mapped a QTL *qCWA9.1* located on ChrA09 using sister-line hybridization and BSA technology (**Figure 3**, **Supplementary Figure S2c**). We further reduced the QTL interval, which is now flanked by markers 9AS337 and 9AS339, and was located at nucleotides 64,453,151 and 64,502,156, respectively (**Figure 4**). Based on these findings, we suggest that a novel candidate gene may be involved in cuticular wax synthesis in *Brassica napus.*

Nine candidate genes were predicted based on the fine mapping of *qCWA9.1* in a 49-Kb interval, and *BnaA09G0695600ZS*, which encodes the cytochrome P450 protein CYP86A4, was identified as one of candidate gene by gene ontology (GO), sequence analyses, expression patterns, and conserved domains prediction. *BnaCYP86A4* is a homologue of *AtCYP86A4* (*AT1G01600*), which was identified as a ω-hydroxylase involved in cutin biosynthesis in *Arabidopsis* (Li-Beisson et al., 2009; Rupasinghe et al., 2007). In this study, we identified *BnaCYP86A4* as the candidate gene associated with cuticular wax biosynthesis. Additionally, another copy of *CYP86A4*, *BnaA10g00380D*, in *Brassica napus* has also been as one of candidate genes was involved in leaf cuticular wax synthesis (Jin et al., 2020; Long et al., 2023). Similarly, *CYP86A4* in apple and *CYP86* (*TraesCS6B03G0695700*) in wheat have been reported as candidate genes related to cuticular wax biosynthesis (Keller-Przybyłkowicz et al., 2025; Wen et al., 2021).Additionally, a comprehensive physiological, transcriptomic and metabolomic analyses of glossy leaf mutants and wild-type rapeseed under drought stress, and uncovered three paralogs of *BnaA09G0695600ZS* (CYP86A4) involved in leaf cuticular wax biosynthesis and drought response (Zhang et al., 2025). Furthermore, recent research has shown that overexpression of *CYP86A7/A8* in poplar, both members of the CYP86 subfamily of P450 enzymes, leads to wax accumulation in leaves, with significantly elevated levels of alkanes (C25, C27, and C28) and primary alcohols (C28 and C29) (Zhao et al., 2026a). Therefore, we strongly propose that *BnaCYP86A4_A09a* (*BnaA09G0695600ZS*) identified in the present study is highly likely to play a role in cuticular wax synthesis. Although BnaA09G696300ZS is an uncharacterized protein within the candidate region and lacks documented functional annotation in the current study, the significant differences observed in BSA-seq variation and transcriptional expression between ‘4074’ and ‘4075’ suggest that it cannot be ruled out as a candidate gene for cuticular wax synthesis. In future research, we will verify the functions of BnaCYP86A4_A09a (BnaA09G0695600ZS) and BnaA09G696300ZS in cuticular wax synthesis using transgenic approaches, including overexpression, gene-edited knockout, and genetic complementation.

Although the expression levels of *BnaCYP86A4_A09a* (*BnaA09G0695600ZS*) were significantly higher in the glossy plant ‘4075’ compared to ‘4074’ at Leaf_4 and Leaf_5 stages, the main components of leaf cuticular wax, including alkanes and primary alcohols, were dramatically decreased in ‘4075’. This contrasts with the phenotype observed in Ox-PtrCYP86A7/A8 plants. This discrepancy may be due to the possible reasons as follows: (1) Although the expression levels of BnaCYP86A4_A09a (BnaA09G0695600ZS) were significantly higher in the glossy plant ‘4075’ compared to ‘4074’ at the Leaf_4 and Leaf_5 stages, the main components of leaf cuticular wax, including alkanes and primary alcohols, were dramatically decreased in ‘4075’. This contrasts with the phenotype observed in Ox-*PtrCYP86A7/A8* plants. This discrepancy may be explained by the following possible reasons: (1) Evolutionary divergence in substrate specificity among CYP86A homologs across plant species. Although PtrCYP86A7/A8 in poplar and BnaCYP86A4_A09a in rapeseed belong to the same CYP86A subfamily, they may have evolved distinct catalytic preferences. In Arabidopsis, CYP86A7/A8 primarily ω-hydroxylate long-chain and very-long-chain fatty acid (VLCFA) precursors that feed directly into alkane and primary alcohol synthesis (Wellesen et al., 2001; Rupasinghe et al., 2007; Li-Beisson et al., 2009), whereas CYP86A4 preferentially metabolizes medium-chain fatty acids (C14:0, C16:0), which are required for the accumulation of C16 cutin monomers in the synthesis of 10,16-dihydroxypalmitate (Li-Beisson et al., 2009). *cyp86a8* mutant plants exhibit severe pleiotropic phenotypes associated with developmental cuticular deficiencies under normal growth conditions (Wellesen et al., 2001). The polyester monomer profile of flowers from *cyp86a4* mutants indicated that C16:0 α, ω-dicarboxylic acids (DCA) and C16:0 fatty acids (FA) were significantly reduced, while C18 DCAs (C18:2, C18:1, C18:0) and C18 FAs (C18:2, C18:1) showed no significant differences (Li-Beisson et al., 2009). Therefore, elevated expression in polar transgenic plants straightforwardly boosts wax precursor flux and increases cuticular wax accumulation. In contrast, significant upregulation of *BnaCYP86A4_A09a* in the glossy line 4075 may competitively sequester shared upstream VLCFA substrates. The diversion of limited precursor pools toward medium-chain lipid metabolism restricts the supply of substrates required for alkane and primary alcohol biosynthesis, ultimately lowering the abundance of these major wax compounds despite high transcript levels of this *CYP* gene. (2) Distinct genetic backgrounds and intact endogenous regulatory networks characterize the poplar *PtrCYP86A7/A8* overexpression lines, which are simple single-gene transgenic lines on a uniform wild-type background. Only one exogenous gene is artificially upregulated, without disturbing other core wax biosynthetic enzymes or repressors. In contrast, although rapeseed lines 4074 and 4075 are derived from the same glossy mutant *39J7H*, line 4075 may harbor widespread genomic variations affecting the entire wax metabolic pathway. For example, key downstream wax biosynthetic genes (e.g., *CER3*, *MAH1*), responsible for alkane and primary alcohol formation, may exhibit intrinsically low transcriptional or enzymatic activity in 4075, preventing the conversion of CYP86A4-derived hydroxyl fatty acids into surface wax constituents. Additionally, ABCG wax transporters, which mediate lipid secretion from epidermal cells to the cuticle, are likely suppressed in 4075, causing intracellular retention of wax intermediates rather than their deposition on leaf surfaces. Furthermore, negative transcriptional repressors of wax biosynthesis may be constitutively activated in 4075, counteracting the high expression of *BnaCYP86A4_A09a* at post-transcriptional, translational, or protein functional levels. (3) High expression of *BnaCYP86A4_A09a* represents a compensatory feedback response rather than a causal driver of increased wax accumulation. The upregulation of *BnaCYP86A4_A09a* is observed only at the Leaf_4 and Leaf_5 stages, suggesting it reflects a physiological feedback mechanism responding to pre-existing wax deficiency instead of initiating wax synthesis. When alkane and primary alcohol levels decline during early seedling stages, leaf epidermal cells activate a compensatory transcriptional circuit that induces CYP86A family wax hydroxylation genes to restore normal cuticular lipid levels. In this context, elevated gene expression is a downstream effect of the glossy phenotype, not its cause. This native feedback regulatory loop is absent in poplar transgenic plants, where artificially enhanced CYP expression directly promotes wax production without homeostatic counter-regulation. (4) Potential differences in protein subcellular localization and tissue expression bias. Constitutively overexpressed PtrCYP86A7/A8 proteins in poplar are stably localized to the endoplasmic reticulum of leaf epidermal cells, the primary site of cuticular wax biosynthesis. In line 4075, sequence variations in the signal peptide of BnaCYP86A4_A09a may lead to partial mis-targeting of the encoded enzyme to mesophyll cells instead of the epidermis. Although whole-leaf qRT-PCR detects significantly higher total transcript abundance, the functional CYP86A enzyme pool within wax-synthesizing epidermal cells remains insufficient to support normal wax loading. Collectively, this discrepancy highlights that the regulatory roles of *CYP86A* subfamily genes in cuticular wax formation may exist species specificity and spatiotemporal specificity in tissue expression, and their functions are tightly constrained by upstream and downstream components of the conserved wax biosynthesis pathway. Of course, these potential causes will be verified in future experiments such as transgenic validation, substrate specificity assays, enzyme activity determination, cutin and cuticular wax components detection, protein-protein interaction studies, and protein-DNA interaction analyses in rapeseed.

### 4.2 Diversity function of CYP86 subfamily members

In this study, we identified a member of the cytochrome P450 family, BnaCYP86A4_A09a, belonging to the CYP86 clan of P450 enzymes. The CYP86 clan is phylogenetically related to plants fatty acid hydroxylases and consists of five families: CYP86, CYP94, CYP96, CYP704, and CYP730 (Nelson, 1999; Nelson et al., 2004). The members of CYP86 clan have diverse catalytic functions. Members of the CYP86A and CYP94 subfamilies have been shown to catalyze fatty acid oxygenation *in vitro* (Kandel et al., 2007; Pinot et al., 1999; Wellesen et al., 2001). Cytochrome P450-dependent hydroxylation is a key step in the formation of C16 and C18 cutin monomers, which are the primary components of cutin and the most abundant extracellular lipids in plant leaves (Höfer et al., 2008; Li-Beisson et al., 2009; Wellesen et al., 2001; Xiao et al., 2004). In current study, phylogenetic analysis revealed that BnaCYP86A4_A09a shared 87.73%, 82.67%, 74.91% amino acid identity with AtCYP86A4, AtCYP86A2 and AtCYP86A8, respectively (**Figure 9b**). Previous studies have shown that AtCYP86A4, AtCYP86A2 and AtCYP86A8 catalyze the ω-hydroxylation of C12 to C18 fatty acids and involve in cutin synthesis. However, some members of CYP86 subfamily, such as CYP96A15 and CYP96B5, have recently been found to catalyze the hydroxylation of alkanes involved in cuticular wax formation (Greer et al., 2007; Zhang et al., 2020). Meanwhile, overexpression of *PtrCYP86A7/A8* in polar led to significant increase in C16-C18 long-chain fatty acids, indicating that there were sufficient precursor substances for cutin and wax biosynthesis (Zhao et al., 2026a). Additionally, wax components analysis showed that the content of C25, C27, and C31 alkanes, as well as C28 and C29 primary alcohols, were significantly elevated in Ox-*PtrCYP86A7/A8* compared to the WT (Zhao et al., 2026a). Therefore, we speculate that *BnaCYP86A4_A09a* may simultaneously possess the functions in both wax and cutin synthesis. Similar to the core shunt mechanism in *Arabidopsis* cuticular wax biosynthesis, where the directional shunting of precursors to either alcohol- or alkane-forming pathways is tightly regulated by the SOH1-CER3-CER1 module in response to environmental conditions (Li et al., 2025). There may be regulatory factors interacting with CYP86A4 that concurrently control the cutin and wax synthesis. To elucidate the specific function and catalytic pathway of *BnaCYP86A4*, we will analyze the content of cutin and cuticular wax in leaves of overexpression-*BnaCYP86A4* lines, loss-of-function *cyp86a4* mutants, and WT plants. Additionally, enzyme activity assays and protein-protein interaction studies will be conducted to verify the molecular regulatory network of *BnaCYP86A4* in cuticular wax biosynthesis.

A comparison of the genomic sequence of *BnaCYP86A4_A09a* between the ‘4074’ and ‘4075’ revealed that the gene consisted of two exons and one intron in both lines (**Figure 6**). In ‘4075’, two SNP substitutions were identified in the first exon, the deduced protein sequence indicated that the 231st SNP resulted in a Lys-to-Asn substitution, whereas the 285th SNP did not cause any amino acid change (**Figure 6**). Although Lys77Asn substitution occurred within the conserved p450 domain, it was not located in critical motifs, such as the heme-binding domain or Thr-containing binding pocket, which are essential for P450 function. Moreover, 3D protein structure prediction suggested that the Lys77Asn substitution leading to the binding sites alteration (**Figure 9c**). Therefore, whether Lys77Asn substitution causes functional changes requires further confirmation.

### 4.3 Variation in cis-regulatory elements potentially affecting expression of *BnaCYP86A4*

*Cis*-regulatory elements encode genomic instructions that govern the precise spatiotemporal patterning of gene expression, which is essential for proper development and an organism’s ability to respond to environmental stimuli. Numerous studies have indicated that promoter variants can significantly affect phenotypic changes (Marand et al., 2023; Wang et al., 2021). In this study, the expression of *BnaCYP86A4_A09a* was significantly elevated in the glossy line ‘4075’. Sequence analysis of *BnaCYP86A4_A09a* indicated that differences in expression levels were likely attributable to variations in the promoter region. Therefore, we turned our attention to the promoter region of *BnaCYP86A4_A09a*. Three SNPs were identified in the promoter region of *BnaCYP86A4_A09a* between the ‘4074’ and ‘4075’ lines. Notably, the mutation at position-378th of *BnaCYP86A4_A09a* in ‘4075’ resulted in the loss of two *cis*-acting elements, the TATC-box and the I-box (**Figure 6**, **Supplementary Figure S5**). These elements commonly appear in plant-induced promoters and are involved in GA, ABA, and light responsiveness (Luo et al., 2013; Nardia et al., 2014; Zhang et al., 2014). Interestingly, phytohormones and light are important internal and external factors that influence cuticular wax synthesis (Yuan et al., 2020; Zhao et al., 2026b).

Notably, some members of the CYP86 clan, OsCYP96B4 and CYP714D1, have been reported to influence lipid metabolism by fine-tuning the GA-to-ABA balance or by regulating gibberellin and ion homeostasis, respectively (Tamiru et al., 2015). Moreover, multiple *cis*-elements response to phytohormones were observed in the promoter of *BnaCYP86A4_A09a*. Consistent with this, *BnaCYP86A4*_*A09a* expression was induced by multiple plant hormones, including IAA, GA, ABA, TZ, JA, and BL, in leaves (**Figure 7a**). Notably, *BnaCYP86A4_A09a* showed a sharp upregulation within 0.5 h of treatment with IAA, GA, JA, BL, ABA, and TZ. Furthermore, ABA and TZ exhibited persistent induction of *BnaCYP86A4_A09a*. Additionally, the expression profile of genes involving in ABA and GA signaling and metabolism indicated that the total level of bioactive GA may not change in the leaves of ‘4075’, whereas the level of ABA was accumulated. Therefore, we hypothesize that the absence of the TATC-box and T-box in the promoter of *BnaCYP86A4* in ‘4075’ alters the response of related genes involved in GA and ABA signaling and metabolism, disrupting the balance between GA and ABA. Continuous accumulation of ABA in ‘4075’ may promote the expression of *BnaCYP86A4*_*A09a*, which in turn disrupts lipid metabolism and alters wax composition, potentially contributing to the glossy phenotype. This hypothesis requires further confirmation through precise measurement of hormone levels, identification of the transcription factors that interact with the TATC-box and T-box, and analysis of the cuticular wax composition of leaves in future studies.

## 5 Conclusion

In this study, through phenotypic observation and genetic analysis, we identified a dominant glossy mutant, *39J7H*. We performed BSA-seq and fine mapping to identify a 49.0-kb QTL, *qCWA9.1*, on ChrA09, which controls cuticular wax synthesis in rapeseed. This was achieved using the homozygous and stable sister lines, ‘4074’ (glaucous) and ‘4075’ (glossy), derived from the offspring of the glossy mutant *39J7H.* Nine genes were included within this interval. Among them, a novel gene *BnaA09G0695600ZS*, encoding the cytochrome P450 protein CYP86A4, was identified as one of candidate genes by cloned sequence analysis, expression patterns, and conserved domains predictions. Clone sequencing revealed SNPs variations in the first exon and promoter region in *BnaCYP86A4_A09a* (*BnaA09G0695600ZS*) between the ‘4074’ (glaucous) and ‘4075’ (glossy) genotypes, which may underlie the differences in waxy phenotype. Further functional validation of *BnaCYP86A4*_*A09a* will help to reveal the genetic basis of cuticular wax biosynthesis in rapeseed. These findings provide new insights and identify a candidate gene for the cuticular wax synthesis pathway in rapeseed, which may be valuable for marker-assisted selection in breeding programs aimed at improving abiotic stress resistance.

## Data Availability

The datasets presented in this study can be found in online repositories. The names of the repository/repositories and accession number(s) can be found in the article/**Supplementary Material**.
