# Supplementary material for: Phenotypic characterization of a dominant glossy mutant and fine mapping of qCWA9.1 for controlling cuticular wax biosynthesis in rapeseed (Brassica napus L.)
Source: Front Plant Sci. 2026 Jul 14;17:1897630. doi: 10.3389/fpls.2026.1897630 (PMC13408256; doi:10.3389/fpls.2026.1897630)
Supplement: Supplementary file 1 [file DataSheet1.docx]

Supplementary Material

Phenotypic characterization of a dominant glossy mutant and fine mapping of *qCWA9.1* for controlling cuticular wax biosynthesis in rapeseed (*Brassica napus* L.)

**Lei Lei, Xirong Zhou, Weirong Wang, Xianmin Meng, Hongru Liu, Hongwei Li, Jifeng Zhu^*^**


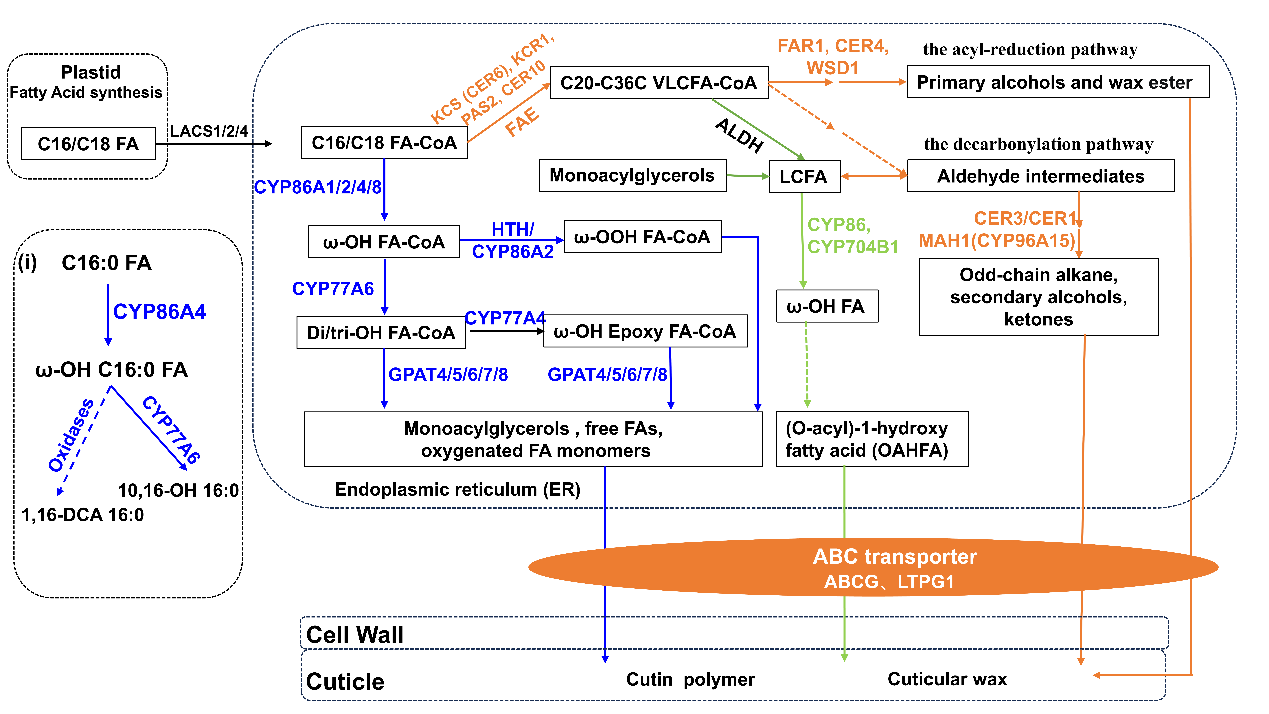


**Supplementary Figure 1.** **The biosynthetic pathways of cutin and cuticular wax, along with the regulatory factors involved in these processes** (Fich et al., 2016; Wen et al., 2021). Blue arrows indicated the pathway of cutin synthesis in *Arabidopsis*, whereas orange arrows indicated the pathway of cuticular wax synthesis. Green arrows indicated the pathway of (O-acyl)-1-hydroxy fatty acid (OAHFA) synthesis in wheat (Wen et al., 2021). OAHFA is a lipid component in wheat leaf epidermis, belong to the cuticular wax. The arrows with solid lines indicated proteins, or protein complexes in the step a demonstrated function, and the arrows with dotted lines indicated no corresponding catalase has been found. Names shown in orange, blue, and green denote proteins involved in the pathway of cutin, cuticular wax and OAHFA synthesis. Abbreviations: C, carbon; CoA, coenzyme A; FA, fatty acid; FAE, fatty acid elongase complex; LCFA, long chain fatty acid.


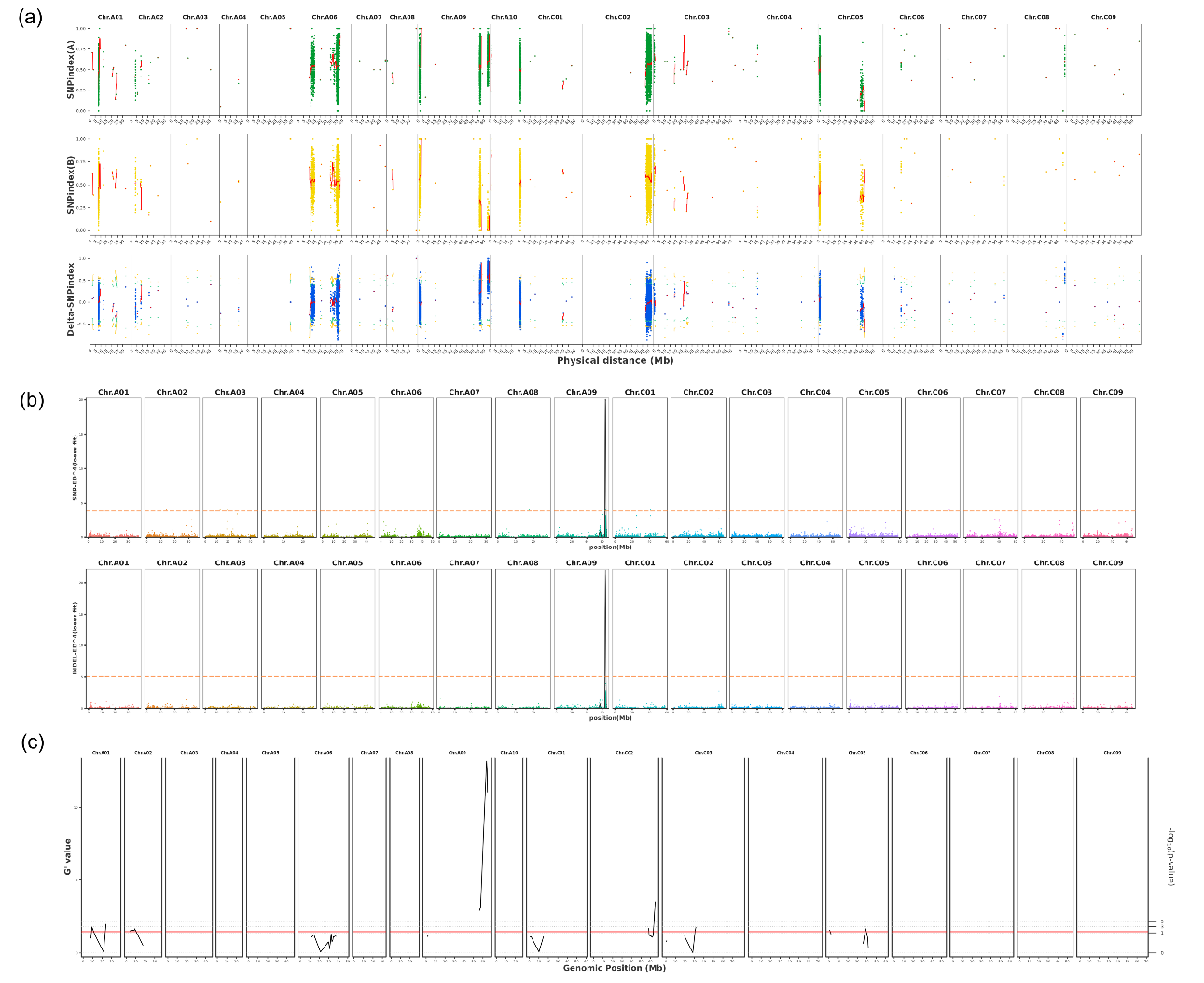
**Supplementary Figure 2.** **Quantitative trait locus (QTL) analysis of rapeseed cuticular wax at leaves using 3 QTL-seq methods.** (a) SNP-index plots of glossy bulk, glaucous bulk, and Δ (SNP-index) between glossy bulk and glaucous bulk. (b) Manhattan plot showing the distribution of Euclidean distance (ED^4) on chromosomes. (c) Manhattan plot showing the distribution of G-value on chromosomes.


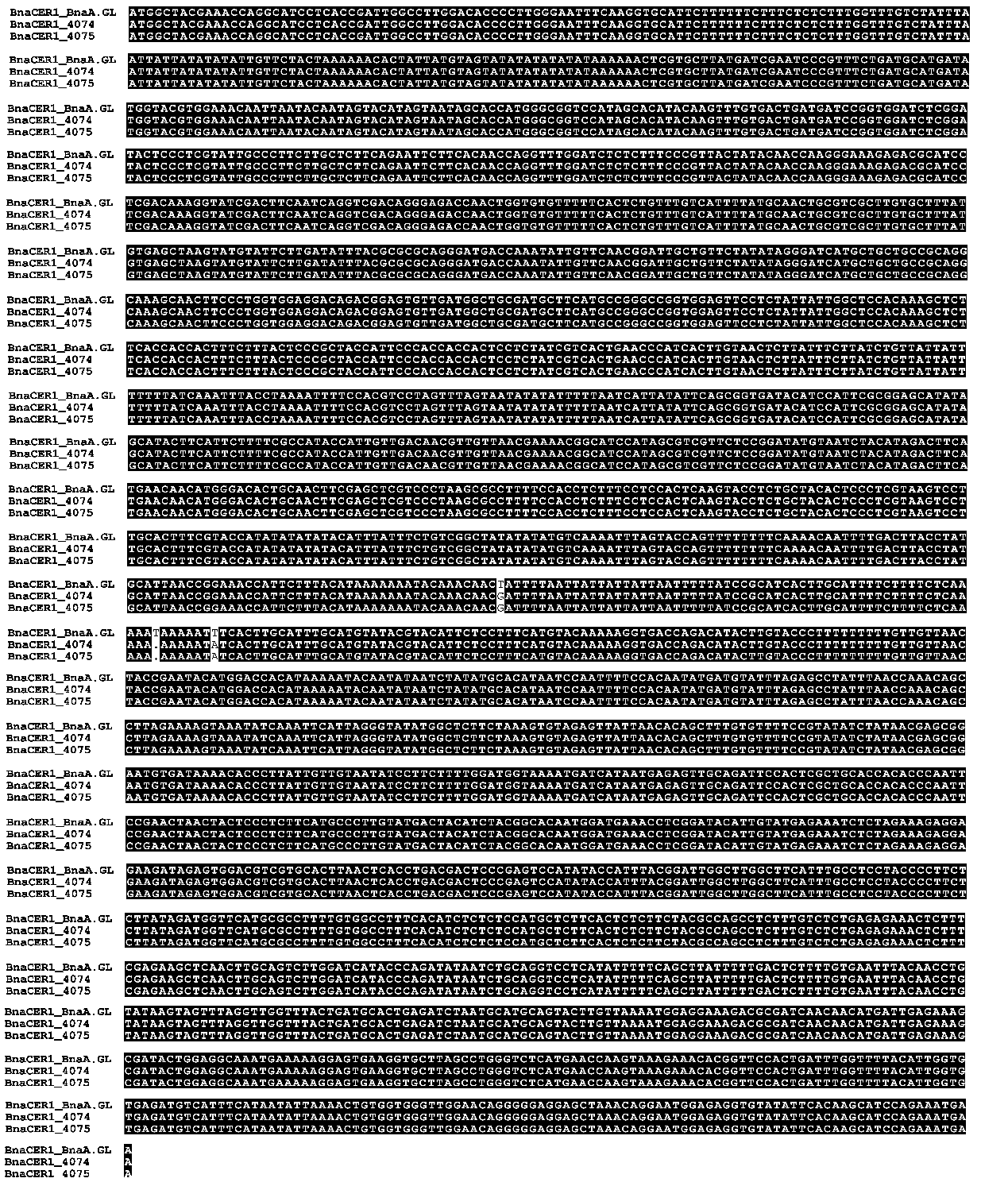


**Supplementary Figure 3**. **Genomic sequences alignment of *BnCER1* in a glossy mutant *BnaA.GL* that reported in previous study (Pu et al., 2013) and in lines ‘4074’ and ‘4075’.**


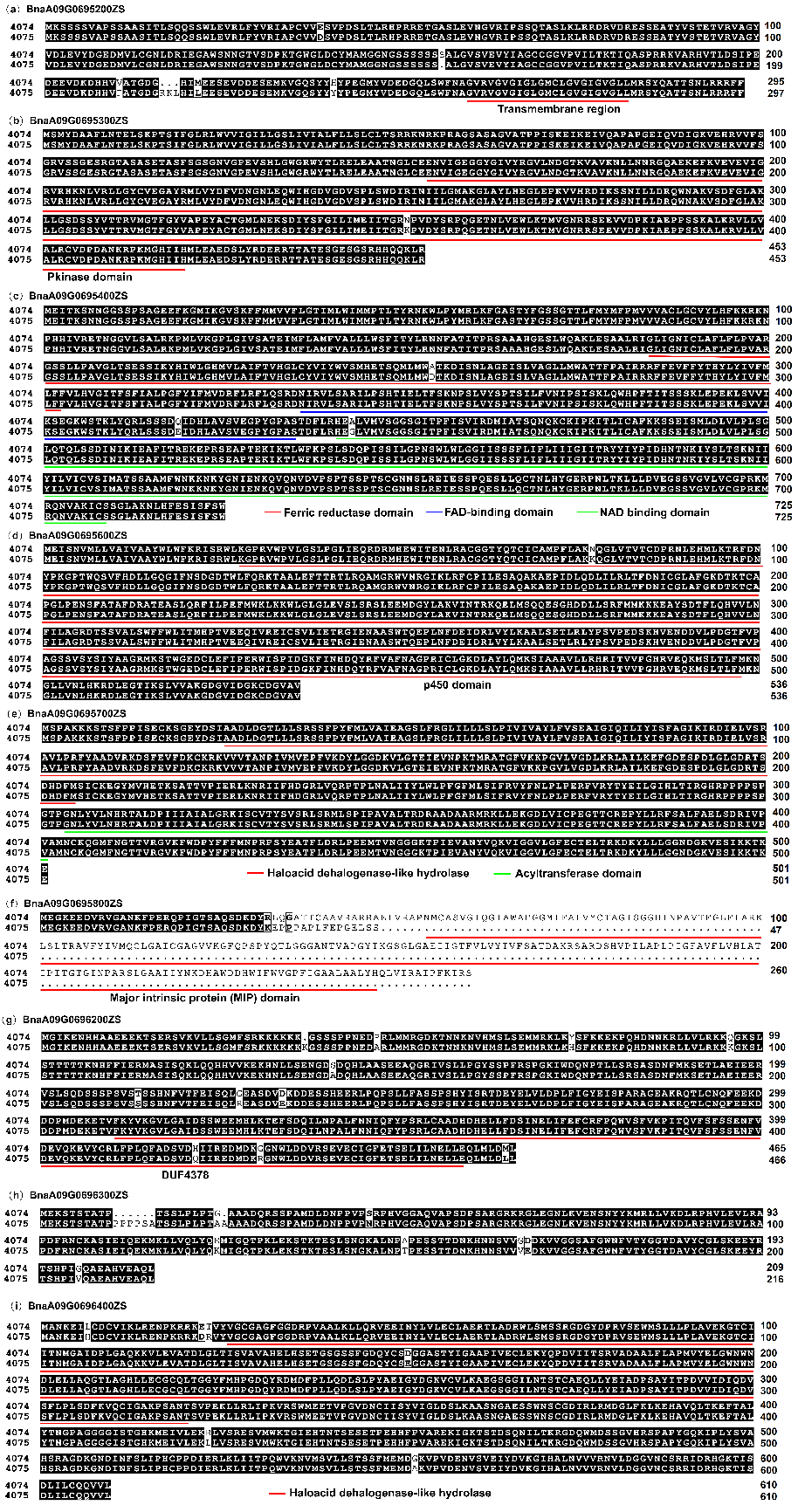
**Supplementary Figure 4. Multiple sequences alignment of the putative protein sequences and conserved domain of nine candidate genes in ‘4074’ and ‘4075’.** The locations of the conserved domains are marked with red, blue, and green lines.


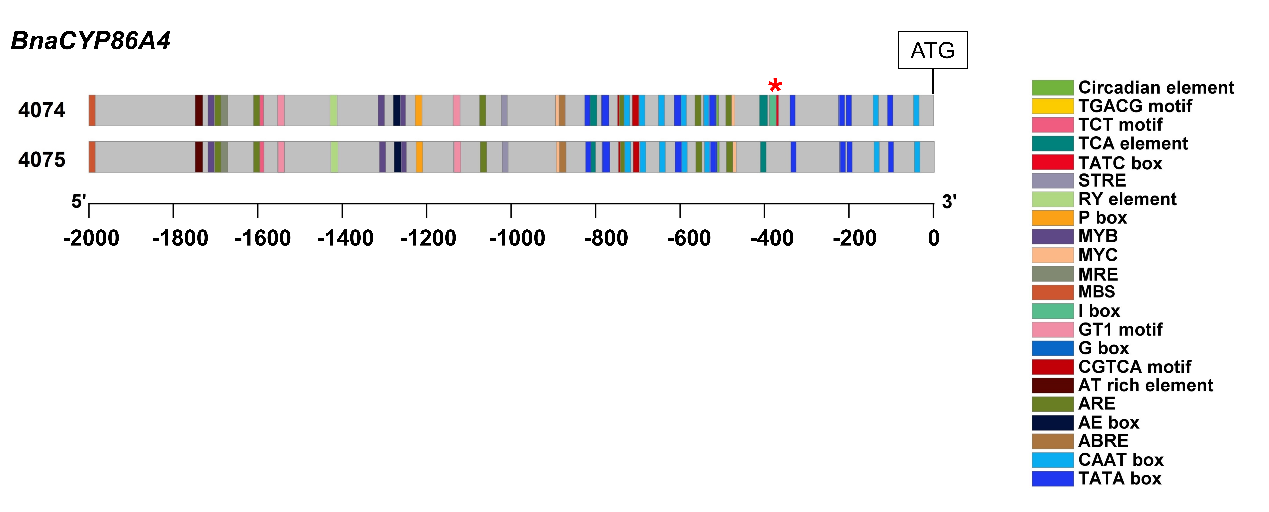


**Supplementary Figure 5. Promoter analysis of** **the *BnaCYP86A4_A09a* (*BnaA09G0695600ZS*) gene in lines ‘4074’ and ‘4075’.** Different *cis*-elements are marked with distinct color symbols at their relative positions within the sequences. The mutation at position -378 bp of the *BnaCYP86A4* promoter in line ‘4075’ resulted in the deletion of two *cis*-elements, I-box and TATC-box (marked with an asterisk).


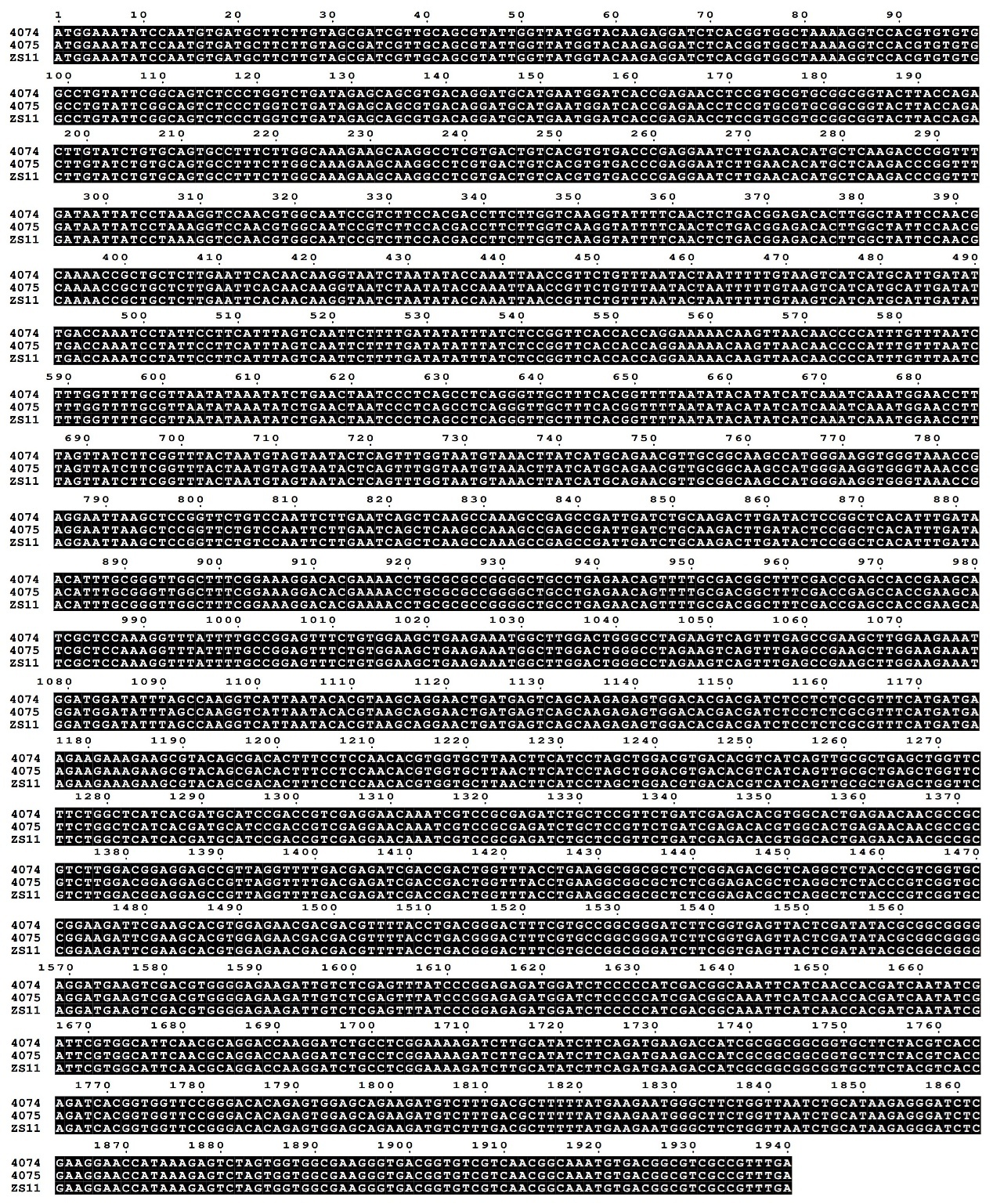


**Supplementary Figure 6. Genomic sequences alignment of *BnaCYP86A4_A09b* (*BnaA09G0717100ZS*) *in* lines ‘4074’, ‘4075’, and ‘ZS11’.**

**Supplementary
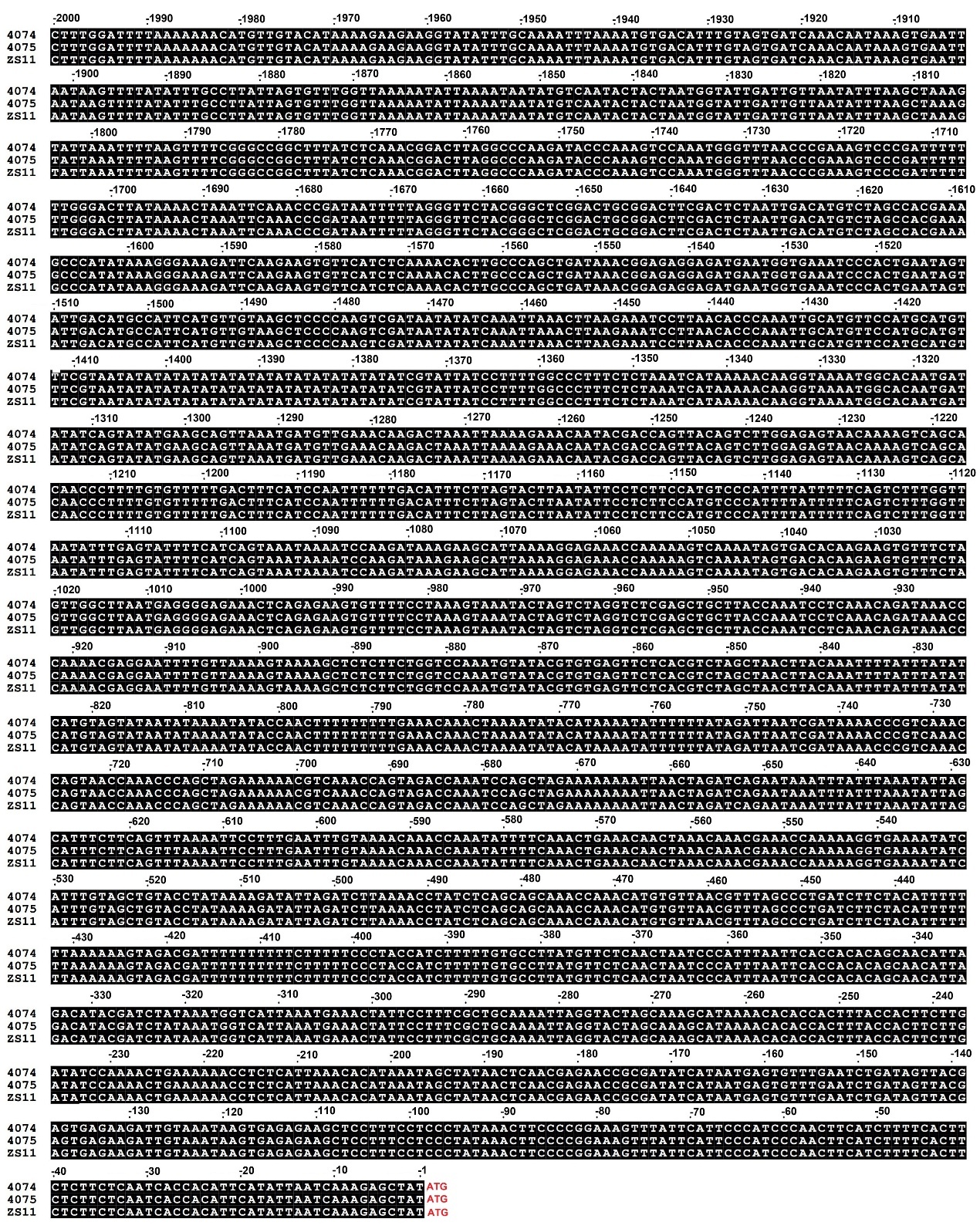
Figure 7. Promoter sequences alignment of *BnaCYP86A4_A09b* (*BnaA09G0717100ZS*) in lines ‘4074’, ‘4075’, and ‘ZS11’.**


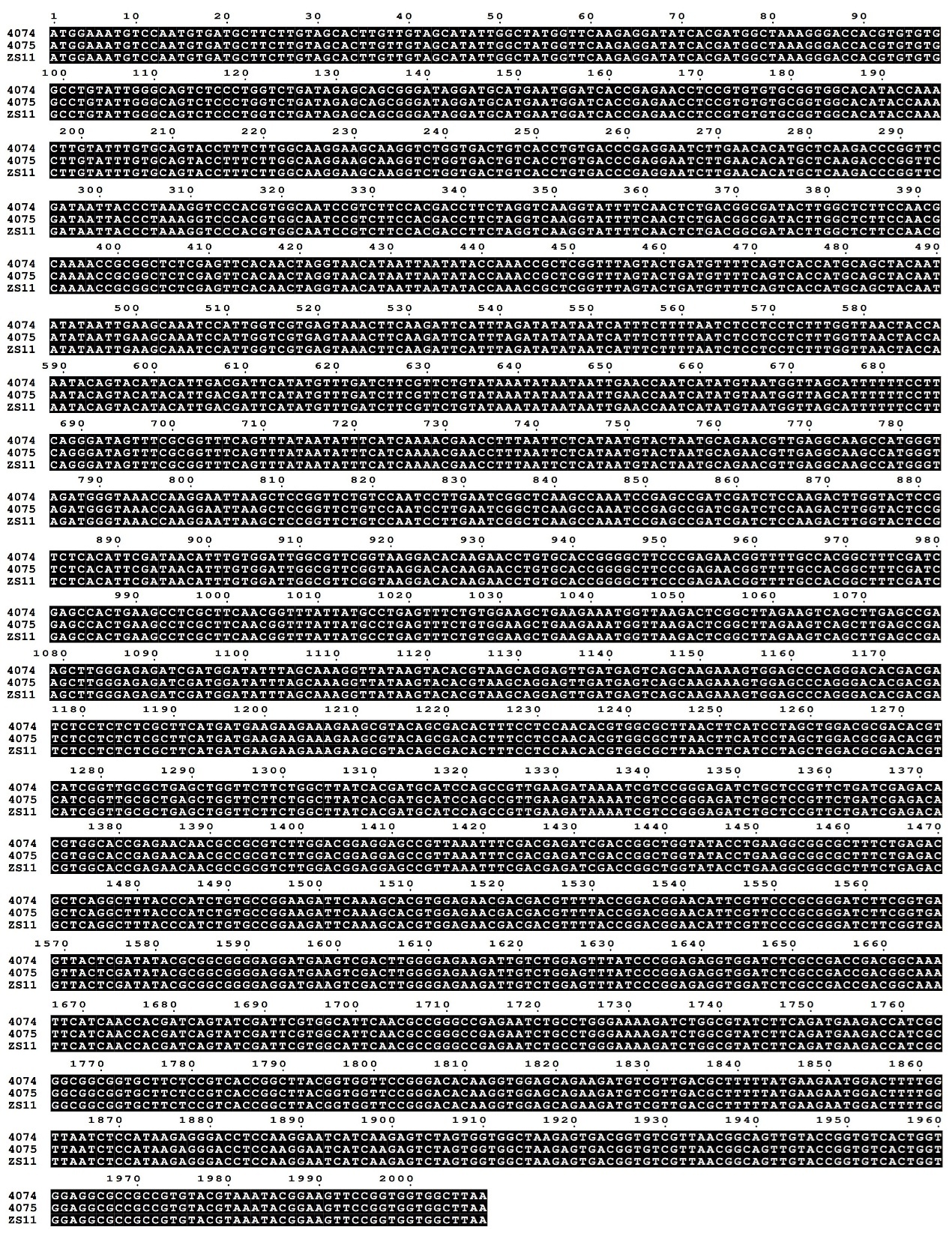


**Supplementary Figure 8. Genomic sequences alignment of *BnaCYP86A4_A10* (*BnaA10G0004100ZS*) in lines ‘4074’, ‘4075’, and ‘ZS11’.**

**Supplementary
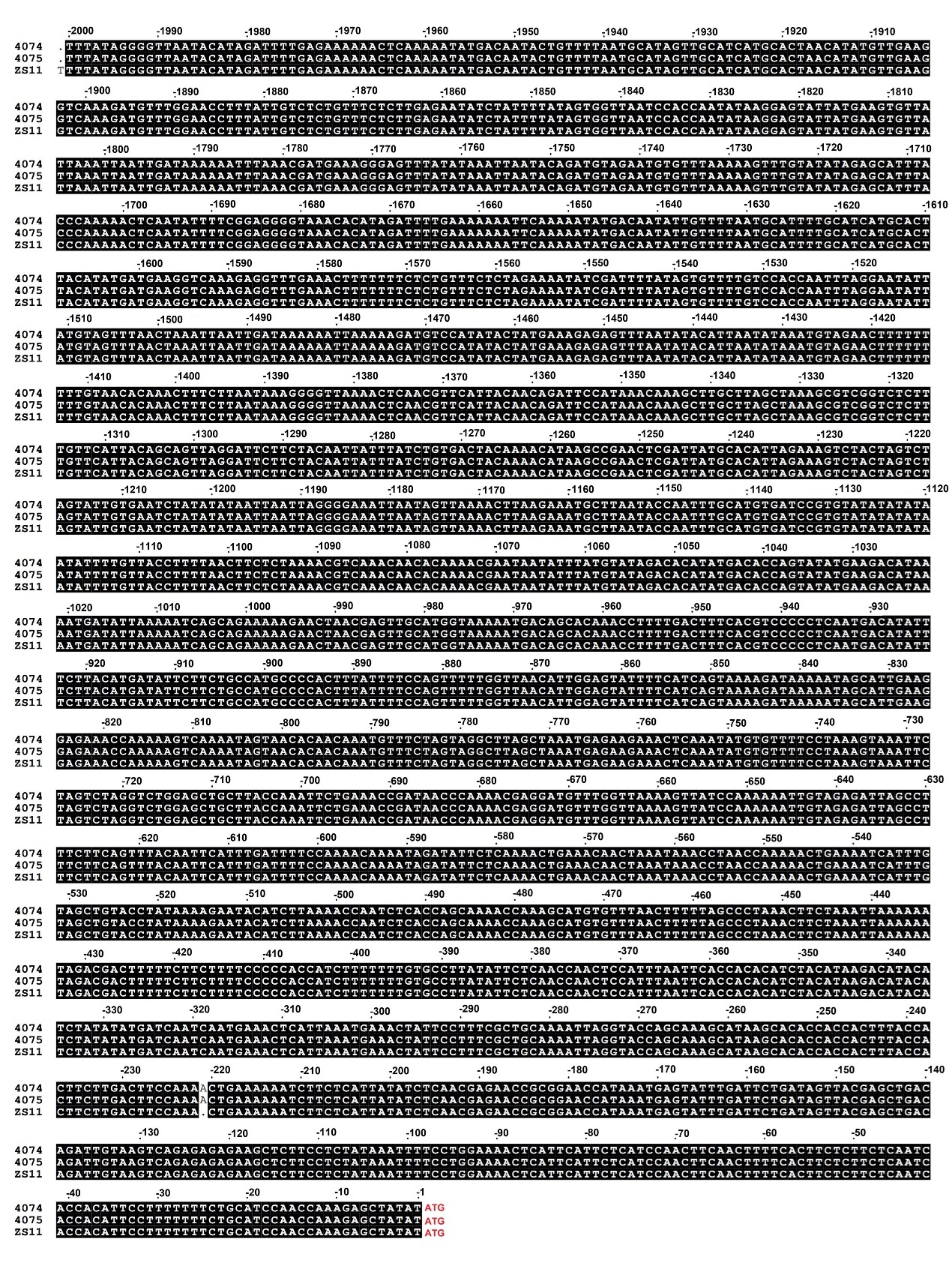
Figure 9. Promoter sequences alignment of *BnaCYP86A4_A10* (*BnaA10G0004100ZS*) in lines ‘4074’, ‘4075’, and ‘ZS11’.**


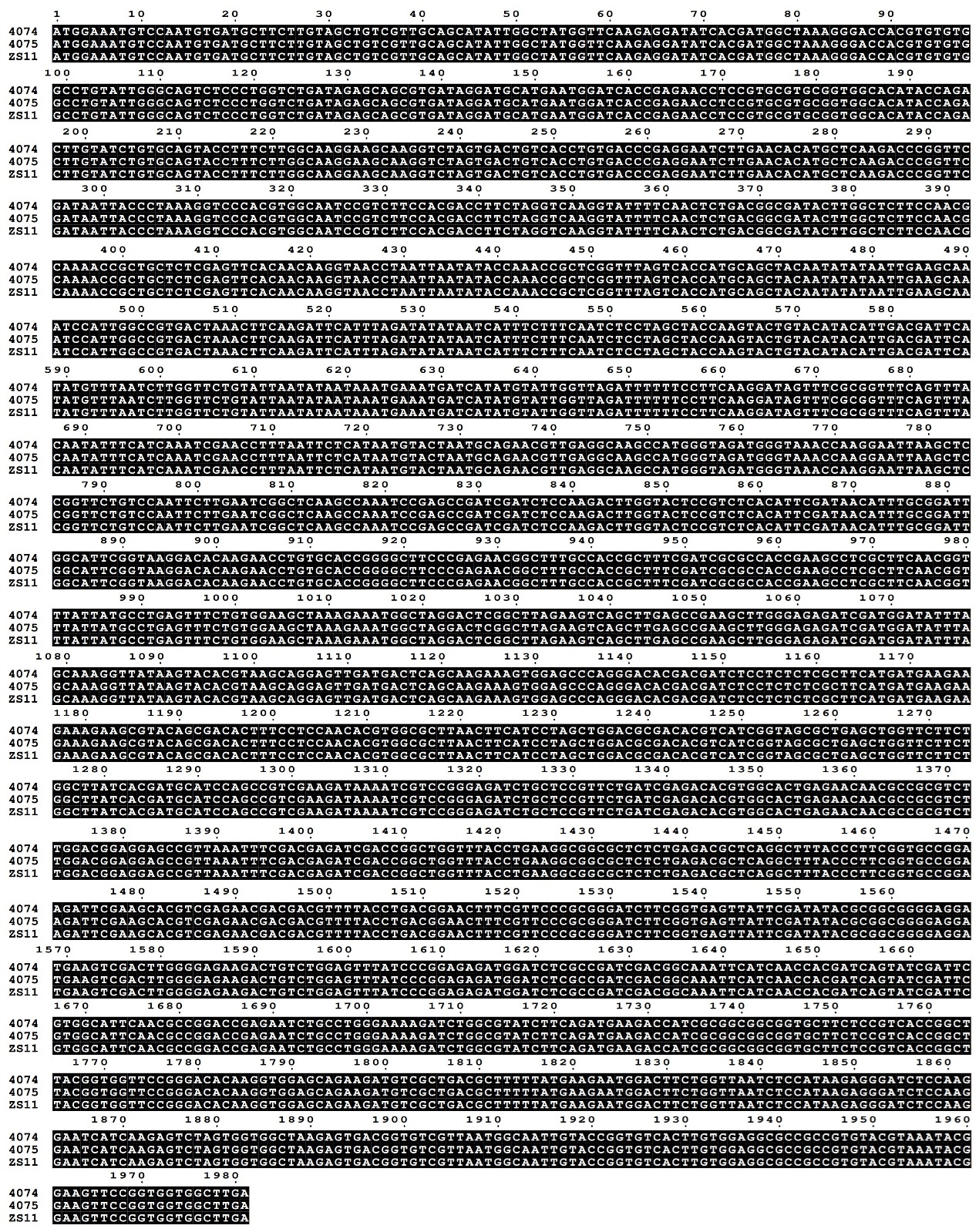


**Supplementary Figure 10. Genomic sequences alignment of *BnaCYP86A4_C05* (*BnaC05G0006000ZS*) in lines ‘4074’, ‘4075’, and ‘ZS11’.**

**Supplementary Figure 11. Promoter sequences alignment of *BnaCYP86A4_C05* (*BnaC05G0006000ZS*) in lines ‘4074’, ‘4075’, and ‘ZS11’.
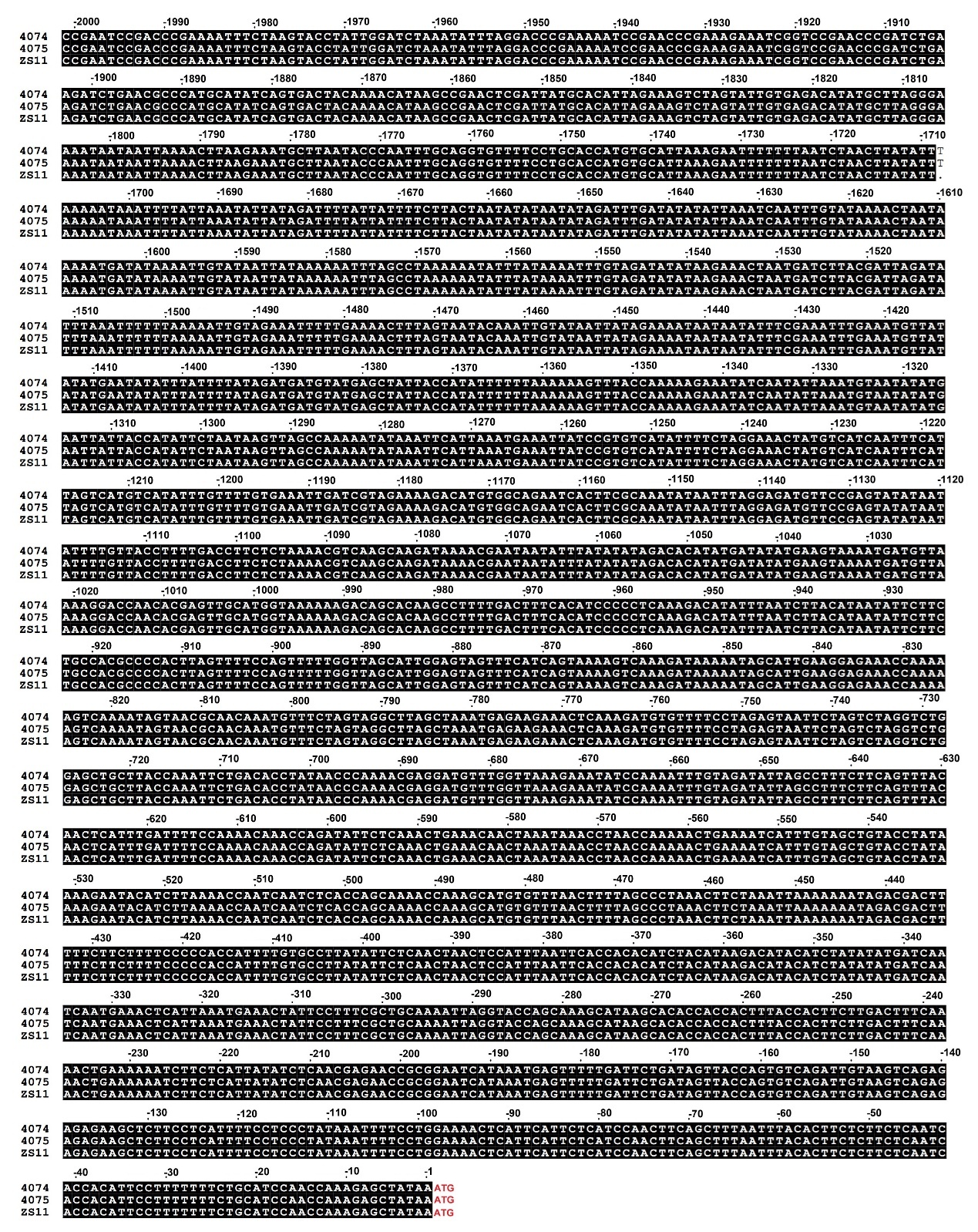
**


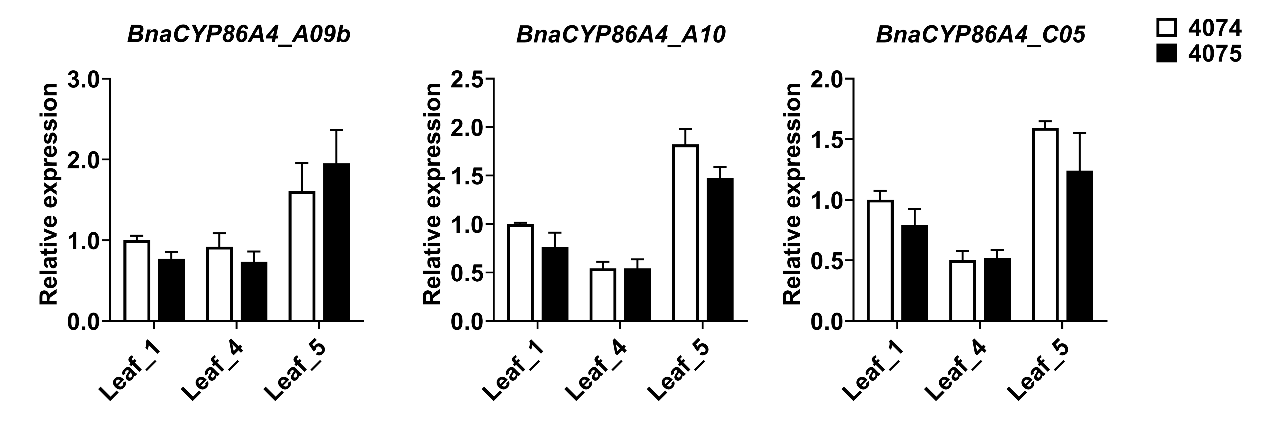


**Supplementary Figure 12. Expression pattern of the other three copies of *BnaCYP86A4s* in lines ‘4074’ and ‘4075’.**

**Table S1. Cuticular wax composition in leaves of ‘4074’ and ‘4075’ plants.**

|  | **4074** | **4075** | ***P* value** |
| --- | --- | --- | --- |
| Total (μg/cm^2^) | 49.6 ± 3.9 | 16.2 ± 1.8 | 0.0004^***^ |
| Alkanes (μg/cm^2^) | 30.7 ± 3.5 | 9.7 ± 1.0 | 0.0013^**^ |
| Esters (μg/cm^2^) | 10.5 ± 1.9 | 3.4 ± 0.6 | 0.0078^**^ |
| Ketones (μg/cm^2^) | 2.8 ± 0.5 | 1.0 ± 0.2 | 0.009^**^ |
| Aldehydes (μg/cm^2^) | 1.1 ± 0.2 | 0.3 ± 0.2 | 0.0138^*^ |
| Secondary alcohols (μg/cm^2^) | 0.4 ± 0.3 | - | - |
| Fatty acids (μg/cm^2^) | 3.2 ± 0.7 | 1.0 ± 0.4 | 0.0207^*^ |
| Primary alcohols (μg/cm^2^) | 1.5 ± 0.5 | 0.4 ± 0.1 | 0.0384^*^ |

The data represents the mean ± SD from three independent replicates, with asterisks ‘*’, ‘**’, and ‘***’ indicating statistical significance at *p* <0.05, *p* <0.01 and *p* <0.001 using Student’s t-test, respectively.

**Table S2. Summary of Illumina sequencing data.**

| Sample ID | Glossy Bulk | Glaucous Bulk | 4074 | 4075 |
| --- | --- | --- | --- | --- |
| HQ Bases | 30025111875 | 29717255176 | 22414777199 | 21292400122 |
| HQ reads | 203089164 | 201047936 | 151787146 | 144156470 |
| Q20 (%) | 98.22 | 98.17 | 98.10 | 98.22 |
| Q30 (%) | 94.79 | 95.59 | 94.54 | 94.79 |
| Mapped ratio (%) | 99.53 | 99.77 | 97.99 | 99.75 |
| Average depth | 22.32X | 22.33X | 16.63X | 16.33X |
| Coverage_ratio_1X (%) | 90.84 | 90.76 | 90.38 | 90.17 |
| Coverage_ratio_4X (%) | 86.24 | 86.15 | 84.65 | 84.60 |
| Coverage_ratio_10X (%) | 75.7 | 75.49 | 63.86 | 65.13 |
| Coverage_ratio_20X (%) | 33.57 | 31.66 | 14.34 | 15.77 |

**Table S3. 136 candidate genes were identified of *qCWA9.1* in the 2.3 Mb region on Chr.A09**

| Gene_ID | Position of the SNP variant | Region | Function annotation |
| --- | --- | --- | --- |
| *BnaA09G0679600ZS* | 63643763 | nonsynonymous SNV | vacuolar protein sorting-associated protein 32 homolog 2-like |
| *BnaA09G0680300ZS* | 63664893,63664896,63664911,63664916,63664929,63664961 | nonsynonymous SNV | beta-hexosaminidase 2 |
| *BnaA09G0681200ZS* | 63751671 | nonsynonymous SNV | glutamate receptor 3.4-like |
| *BnaA09G0681300ZS* | 63757050 | nonsynonymous SNV | 50S ribosomal protein L6 |
| *BnaA09G0681500ZS* | 63763268 | nonsynonymous SNV | -- |
| *BnaA09G0681800ZS* | 63782278,63782328,63782345,63782377,63782422,63782966 | nonsynonymous SNV | glycerol-3-phosphate O-acyltransferase 3/4 |
| *BnaA09G0681900ZS* | 63796100,63797701,63797704,63797821 | nonsynonymous SNV | ARID2; At rich interactive domain 2 (ARID, RFX-like) |
| *BnaA09G0682000ZS* | 63799172,63799538,63799564,63799567,63799882,63799960,63799962,63799976,63800089,63800211, 63800235,63800364, 63800368, 63800391,63800586,63800754,63800788,63800810,  63800875,63801199,63801411,63801628,63801657 | nonsynonymous SNV | ubiquitin carboxyl-terminal hydrolase 2-like |
| *BnaA09G0682100ZS* | 63802780 | nonsynonymous SNV | vesicle-associated membrane protein 721-like |
| *BnaA09G0682200ZS* | 63814894,63814895,63815084 | nonsynonymous SNV | probable pectate lyase 1 |
| *BnaA09G0682300ZS* | 63825624 | synonymous SNV | pollen-preferential protein |
| *BnaA09G0682400ZS* | 63826513 | nonsynonymous SNV | -- |
| *BnaA09G0682500ZS* | 63829612 | synonymous SNV | GRIM-19 protein |
| *BnaA09G0682600ZS* | 63832883 | nonsynonymous SNV | probable indole-3-pyruvate monooxygenase YUCCA3 |
| *BnaA09G0682700ZS* | 63842158,63842566,63843008,63843609,63843683 | nonsynonymous SNV | Pentatricopeptide repeat-containing protein |
| *BnaA09G0682800ZS* | 63844146 | synonymous SNV | AWPM-19-like family |
| *BnaA09G0682900ZS* | 63846527,63848197,63848200,63848211,63848224,63848231,63848245 | nonsynonymous SNV | auxin-responsive protein IAA12-like |
| *BnaA09G0683000ZS* | 63850490 | synonymous SNV | cyclin-D4-1-like |
| *BnaA09G0683100ZS* | 63856818,63857085 | nonsynonymous SNV | galactinol synthase 6-like |
| *BnaA09G0683200ZS* | 63866413,63868244,63868456 | nonsynonymous SNV | Plant family of unknown function (DUF810) |
| *BnaA09G0683300ZS* | 63871191 | nonsynonymous SNV | CRIB domain-containing protein RIC3 |
| *BnaA09G0683400ZS* | 63889215,63889636,63889664,63890868 | nonsynonymous SNV | casein kinase I-like |
| *BnaA09G0683600ZS* | 63902280 | unknown | RING/U-box superfamily protein |
| *BnaA09G0683700ZS* | 63906884,63906910,63907471 | nonsynonymous SNV | 2,4-dihydroxy-1,4-benzoxazin-3-one-glucoside dioxygenase |
| *BnaA09G0683800ZS* | 63909824,63909899,63910052,63910054,63910070,63910096 | nonsynonymous SNV | pre-mRNA-processing factor 39-like |
| *BnaA09G0684000.1ZS* | 63915200,63918745,63919105,63919249,63919254,63919633,63919678 | nonsynonymous SNV | ubiquitin carboxyl-terminal hydrolase 7 |
| *BnaA09G0684100ZS* | 63920840 | synonymous SNV | 40S ribosomal protein S15-1 |
| *BnaA09G0684200ZS* | 63930394 | synonymous SNV | auxin-responsive protein IAA17 |
| *BnaA09G0684300ZS* | 63941210,63941363，63941779 | nonsynonymous SNV | auxin-responsive protein IAA3 |
| *BnaA09G0684400ZS* | 63942323,63942466,63942950,63943398,63943425,63943436,63943602,63943814,63943989,63944196,63944390,63944394,63944417,63944422,63945057,63945075,63945078,63945085,63945091,63945104,63945652,63945689,63945703,63946574,63946724,63946728 | nonsynonymous SNV | protein kinase domain containing protein |
| *BnaA09G0684600ZS* | 63949656,63950102 | nonsynonymous SNV | probable indole-3-pyruvate monooxygenase YUCCA9 |
| *BnaA09G0684700ZS* | 63957553 | synonymous SNV | eukaryotic translation initiation factor 2 subunit gamma-like |
| *BnaA09G0684800ZS* | 63960654 | synonymous SNV | myosin-8-like |
| *BnaA09G0684900ZS* | 63971796,63971905,63971913,63971955,63971966,63972037,63972149,63972155,63972318,63972372,63972381,63972566,63972641,63972785,63973920 | nonsynonymous SNV | E3 ubiquitin-protein ligase Nedd-4 |
| *BnaA09G068500ZS0* | 63975604,63975607,63975608 | nonsynonymous SNV | Activating molecule in BECN1-regulated autophagy protein 1 |
| *BnaA09G0685000.2ZS* | 63977069 | nonsynonymous SNV | Activating molecule in BECN1-regulated autophagy protein 1 |
| *BnaA09G0685100ZS* | 63980122,63980133,63980224 | nonsynonymous SNV | 40S ribosomal protein S7 |
| *BnaA09G0685200ZS* | 63982138,63982885,63983115,63983116,63983377,63983663,63983677,63984036 | nonsynonymous SNV | TATA box-binding protein-associated factor RNA polymerase I subunit B |
| *BnaA09G0685300ZS* | 63986188,63986189,63986255,63986265,63986276,63986721,63986725,63986731,63986742 | nonsynonymous SNV | Large subunit ribosomal protein L10e |
| *BnaA09G0685400ZS* | 63988547,63988549,63988550,63988560,63988571,63988593 | nonsynonymous SNV | Protein JASON |
| *BnaA09G0685600ZS* | 63993709,63995724,63995750 | nonsynonymous SNV | Phospholipid: diacylglycerol acyltransferase |
| *BnaA09G0685800ZS* | 64002262,64002741,64002776 | nonsynonymous SNV | CRIB domain-containing protein RIC8 |
| *BnaA09G0685900ZS* | 64007378 | nonsynonymous SNV | casein kinase I |
| *BnaA09G068600ZS0* | 64015539,64015558,64015611,64015613,64015645 | nonsynonymous SNV | Defensin-like protein 153 |
| *BnaA09G0686100ZS* | 64018177,64018211 | nonsynonymous SNV | Defensin-like protein 153 |
| *BnaA09G0686300ZS* | 64033840 | nonsynonymous SNV | prohibitin-2, mitochondrial-like |
| *BnaA09G0686600ZS* | 64047408 | nonsynonymous SNV | Zinc finger protein MATPIE |
| *BnaA09G0686800ZS* | 64060300,64061062,64061065,64061372,64061378,64063231,64065789 | nonsynonymous SNV | DNA excision repair protein ERCC-6-like 2 |
| *BnaA09G0687100ZS* | 64077507,64078056,64078234 | nonsynonymous SNV | 8-amino-7-oxononanoate synthase |
| *BnaA09G0687200ZS* | 64078659 | nonsynonymous SNV | thioredoxin M-type, chloroplastic |
| *BnaA09G0687300ZS* | 64080195 | nonsynonymous SNV | -- |
| *BnaA09G0687400ZS* | 64081183,64081195,64081234,64081244 | nonsynonymous SNV | -- |
| *BnaA09G0687500ZS* | 64082368,64084266,64084280 | nonsynonymous SNV | secretory carrier-associated membrane protein 2-like |
| *BnaA09G0687600ZS* | 64087050,64087059,64087093,64087095,64087102,64087122, | nonsynonymous SNV | coproporphyrinogen-III oxidase 1, chloroplastic-like |
| *BnaA09G0687800ZS* | 64091546,64091902,64092036,64092079 | nonsynonymous SNV | Protein NETWORKED 3A |
| *BnaA09G0687900ZS* | 64092467,64092992,64092996,64092997,64093080,64093092,64093191 | nonsynonymous SNV | putative DUF21 domain-containing protein |
| *BnaA09G0688000ZS* | 64094540,64094555 | nonsynonymous SNV | putative DUF21 domain-containing protein |
| *BnaA09G0688100ZS* | 64096929,64096950,64099255 | nonsynonymous SNV | transmembrane protein 64 |
| *BnaA09G0688200ZS* | 64101323,64101359,64101554,64102106,64102124,64102127,64102139,64102259,64102349,64102359 | nonsynonymous SNV | Basic 7S globulin |
| *BnaA09G0688300ZS* | 64111325 | synonymous SNV | methylcrotonoyl-CoA carboxylase subunit alpha, mitochondrial |
| *BnaA09G0688700ZS* | 64153375,64153543,64153964,64153965 | nonsynonymous SNV | (+)-neomenthol dehydrogenase |
| *BnaA09G0688800ZS* | 64156358,64156395,64156424,64156480,64156812,64157312,64157313,64157333,64157376,64158182 | nonsynonymous SNV | peroxisome biogenesis protein 6-like |
| *BnaA09G0688900.1ZS* | 64161028,64161563,64161672,64162273,64162437,64162537,64163260 | nonsynonymous SNV | bidirectional sugar transporter SWEEt12-like |
| *BnaA09G0689000ZS* | 64166413,64166433,64166541,64167203,64167598,64168165,64168535,64168544 | nonsynonymous SNV | cullin-1-like |
| *BnaA09G0689100ZS* | 64170582,64170594,64170733,64170741,64170821,64170861,64170872,64170889,64170892,64170896,64170901,64170908,64170938,64170953,64171779 | nonsynonymous SNV | wee1-like protein kinase |
| *BnaA09G0689200ZS* | 64176450 | nonsynonymous SNV | glutathione S-transferase F4-like |
| *BnaA09G0689300ZS* | 64179637,64179729,64179732,64179831,64179832,64179864,64184628,64184897,64185170 | nonsynonymous SNV | elongation factor 1-alpha |
| *BnaA09G0689400ZS* | 64185288,64185665 | nonsynonymous SNV | E3 ubiquitin-protein ligase BAH1-like |
| *BnaA09G0689500ZS* | 64188862,64189117 | nonsynonymous SNV | E3 ubiquitin-protein ligase BAH1-like |
| *BnaA09G0689600ZS* | 64193203,64193211,64193235,64193454,64193480,64193511,64193513,64193550,64193552 | nonsynonymous SNV | Protein of unknown function, DUF538 |
| *BnaA09G0689700ZS* | 64195502,64195508,64195511,64195527,64195541,64195566,64197045,64197067,64197087 | nonsynonymous SNV | endoglucanase 23-like |
| *BnaA09G0689800ZS* | 64202644 | synonymous SNV | 60S ribosomal protein L19-1-like |
| *BnaA09G0689900ZS* | 64204635,64204810,64204875,64205581,64205637 | nonsynonymous SNV | protein DEHYDRATION-INDUCED 19 homolog 2-like |
| *BnaA09G0690000ZS* | 64206520,64206521 | nonsynonymous SNV | protein MRt2-like; mortality factor 4-like protein 1 |
| *BnaA09G0690100ZS* | 64209337,64209582,64209726,64209778,64209811,64209949 | nonsynonymous SNV | probable galacturonosyltransferase 6-like |
| *BnaA09G0690300ZS* | 64217773,64217807,64217815,64218156,64218206,64218207,64218795,64218796,64218802 | nonsynonymous SNV | Lipase |
| *BnaA09G0690400ZS* | 64222171,64223678,64226264,64226324 | nonsynonymous SNV | histone-lysine N-methyltransferase MEDEA-like |
| *BnaA09G0690500ZS* | 64234667 | synonymous SNV | cytochrome P450 78A6 |
| *BnaA09G0691000ZS* | 64258380 | nonsynonymous SNV | polygalacturonase; galacturan 1,4-alpha-galacturonidase |
| *BnaA09G0691100ZS* | 64265025 | nonsynonymous SNV | Protein NIM1-INtERACtINt 1 |
| *BnaA09G0691200ZS* | 64266405 | nonsynonymous SNV | ADP-ribosylation factor 1-like |
| *BnaA09G0691300ZS* | 64267244 | synonymous SNV | cytochrome c oxidase assembly protein COX11, mitochondrial-like |
| *BnaA09G0691500ZS* | 64275276,64275496 | nonsynonymous SNV | -- |
| *BnaA09G0692100ZS* | 64297608,64297961,64297974,64297982,64298031,64298219,64298258 | nonsynonymous SNV | F-box/kelch-repeat protein SKIP6 |
| *BnaA09G0692200ZS* | 64300013,64300030,64300133,64300431,64300622,64300766,64301319 | nonsynonymous SNV | probable serine/threonine-protein kinase |
| *BnaA09G0692300ZS* | 64307683,64308313,64308477,64308550,64308960 | nonsynonymous SNV | hypothetical protein; K18729 protein angel (A) |
| *BnaA09G0692400ZS* | 64310445,64310667 | nonsynonymous SNV | putative plasma membrane phosphate transporter pho87 protein |
| *BnaA09G0692500ZS* | 64313156,64313561,64313596,64313734 | nonsynonymous SNV | NAC domain-containing protein 89-like |
| *BnaA09G0692600ZS* | 64321399 | synonymous SNV | soluble inorganic pyrophosphatase |
| *BnaA09G0692700ZS* | 64326847,64329563 | nonsynonymous SNV | probable fructose-bisphosphate aldolase 3, chloroplastic |
| *BnaA09G0692800ZS* | 64333707,64333723,64333734,64335259 | nonsynonymous SNV | probable fructose-bisphosphate aldolase 3, chloroplastic |
| *BnaA09G0692900ZS* | 64341950,64342197,64342198 | nonsynonymous SNV | pyruvate dehydrogenase E1 component subunit alpha-3, chloroplastic-like |
| *BnaA09G0693100ZS* | 64346009 | synonymous SNV | 3-ketoacyl-CoA synthase |
| *BnaA09G0693200ZS* | 64354623 | synonymous SNV | 5'-AMP-activated protein kinase, catalytic alpha subunit |
| *BnaA09G0693300ZS* | 64360441 | synonymous SNV | -- |
| *BnaA09G0693400ZS* | 64362159 | nonsynonymous SNV | SS18; synovial sarcoma translocation, chromosome 18; protein SSXT |
| *BnaA09G0693500ZS* | 64370996,64371822,64371948,64371975,64372215,64372451 | nonsynonymous SNV | Cytochrome P450 78A6 |
| *BnaA09G0693600ZS* | 64381457 | synonymous SNV | DNA-directed RNA polymerase III subunit RPC10 |
| *BnaA09G0693900ZS* | 64385489,64385737 | nonsynonymous SNV | -- |
| *BnaA09G0694000ZS* | 64385946 | synonymous SNV | bifunctional fucokinase/fucose pyrophosphorylase |
| *BnaA09G0694100ZS* | 64386890 | synonymous SNV | -- |
| *BnaA09G0694200ZS* | 64387943 | synonymous SNV | ormdl proteins; oligosaccharyltransferase complex subunit delta (ribophorin II) |
| *BnaA09G0694300ZS* | 64390310,64390334,64390472,64390622,64390625,64390636,64390637,64391073,64391180,64391228 | nonsynonymous SNV | -- |
| *BnaA09G0694500ZS* | 64397484,64397535,64397583,64397603,64397671,64397677,64397849,64397894,64398196,64398202,64398322,64398643, | nonsynonymous SNV | Cytochrome P450 703A2 |
| *BnaA09G0694600ZS* | 64398985,64399000,64399014,64399229 | nonsynonymous SNV | cyclic pyranopterin monophosphate synthase accessory protein, mitochondrial |
| *BnaA09G0694700ZS* | 64400512,64400606 | nonsynonymous SNV | pathogenesis-related protein 1-like |
| *BnaA09G0694800ZS* | 64404252 | synonymous SNV | CENH3; histone H3-like centromeric protein HtR12 |
| *BnaA09G0695000ZS* | 64410845 | nonsynonymous SNV | mitogen-activated protein kinase 7 |
| *BnaA09G0695100ZS* | 64427782 | nonsynonymous SNV | FK506-binding protein 4-like |
| *BnaA09G0695200ZS* | 64453305,64453819,64453929,64453986 | nonsynonymous SNV | Erythronate-4-phosphate dehydrogenase family protein |
| *BnaA09G0695300ZS* | 64457574 | nonsynonymous SNV | probable serine/threonine-protein kinase |
| *BnaA09G0695400ZS* | 64462787,64463370,64463540 | nonsynonymous SNV | noxA; superoxide-generating NADPH oxidase flavocytochrome |
| *BnaA09G0695600ZS* | 64473858 | nonsynonymous SNV | cytochrome P450 86A4; fatty acid omega-hydroxylase |
| *BnaA09G0695700ZS* | 64478521 | synonymous SNV | glycerol-3-phosphate 2-O-acyltransferase 4 |
| *BnaA09G0695800ZS* | 64480368 | synonymous SNV | aquaporin PIP1-3 |
| *BnaA09G0696200ZS* | 64498137,64498218,64498284,64498419,64498741,64498782,64498802,64499569,64499594,64499696 | nonsynonymous SNV | -- |
| *BnaA09G0696300ZS* | 64500222,64500282,64500689,64500767,64500816,64500820,64500933 | nonsynonymous SNV | -- |
| *BnaA09G0696400ZS* | 64502772,64502899,64502901,64503363,64503588,64505402,64505963 | nonsynonymous SNV | phosphatidylcholine-retinol O-acyltransferase |
| *BnaA09G0696700ZS* | 64510608,64511474,64511522,64511574,64511577,64511614,64512105,64514304 | nonsynonymous SNV | K(+) efflux antiporter 1 |
| *BnaA09G0696800ZS* | 64520888 | synonymous SNV | peroxisomal membrane protein 11D |
| *BnaA09G0696900ZS* | 64525147,64525360,64525483,64525522,64525713,64525741,64525770,64525771,64525912,64525939,64526067,64526138,64526185,64526608,64526674,64526706,64526733 | nonsynonymous SNV | flap endonuclease GEN-like 1 |
| *BnaA09G0697000ZS* | 64528336 | synonymous SNV | ATPase GET3 |
| *BnaA09G0697100ZS* | 64530321,64530322,64530741,64530776,64531101,64531112,64532672,64532855 | nonsynonymous SNV | Ribosomal lysine N-methyltransferase set10 |
| *BnaA09G0697200ZS* | 64534561,64534596,64535421,64535425, | nonsynonymous SNV | Ankyrin repeat and zinc finger domain-containing protein 1 |
| *BnaA09G0697300ZS* | 64536239,64536240 | nonsynonymous SNV | Peptidyl-prolyl cis-trans isomerase CYP18-1 |
| *BnaA09G0697400.2ZS* | 64540304,64540345,64540526 | nonsynonymous SNV | Armadillo repeat-containing kinesin-like protein 2 |
| *BnaA09G0697500ZS* | 64543157,64543469 | nonsynonymous SNV | Brefeldin A-inhibited guanine nucleotide-exchange protein 3 |
| *BnaA09G0697600ZS* | 64557813 | synonymous SNV | Protein transport Sec1a |
| *BnaA09G0697800ZS* | 64564112,64565226 | nonsynonymous SNV | Squamosa promoter-binding-like protein 8 |
| *BnaA09G0697900ZS* | 64567783,64568179 | nonsynonymous SNV | -- |
| *BnaA09G0698000ZS* | 64571749,64572165,64574110,64581266 | nonsynonymous SNV | CCR4-NOT transcription complex subunit 1 |
| *BnaA09G0698100ZS* | 64588431 | nonsynonymous SNV | COP9 signalosome complex subunit 7-like |
| *BnaA09G0698200ZS* | 64589281 | synonymous SNV | tRNA wybutosine-synthesizing protein 4 |
| *BnaA09G0698400ZS* | 64598124,64598536,64598852 | nonsynonymous SNV | alpha-1,6-mannosyltransferase |
| *BnaA09G0701600ZS* | 64752898 | nonsynonymous SNV | probable pectinesterase 8 |
| *BnaA09G0701900ZS* | 64762333 | synonymous SNV | ubiquitin-like modifier-activating enzyme 5 |

Note: White bars in the table indicate the gene occurring non-synonymous single nucleotide variants (SNVs) while grey bars in the table indicate the gene occurring synonymous SNVs.

**Table S4. Mutation type analysis of seven candidate genes.**

| Genes | Indel variant in 4075 | | SNP variant in 4075 | | Number of amino acid changes |
| --- | --- | --- | --- | --- | --- |
|  | Location of variant | Variant type | Location of variant | Variant type |  |
| *BnaA09G0695200ZS* | exon1 | frameshift deletion, frameshift insertion | exon1 & exon2 | nonsynonymous SNV | 8 |
| *BnaA09G0695300ZS* | - | - | exon1 & exon5 | nonsynonymous SNV | 1 |
| *BnaA09G0695400ZS* | - | - | exon5 & exon6 & exon7 | nonsynonymous SNV | 3 |
| *BnaA09G0695600ZS* | - | - | exon1 | nonsynonymous SNV | 1 |
| *BnaA09G0695700ZS* | - | - | exon1 | synonymous SNV | 0 |
| *BnaA09G0695800ZS* | exon1 | frameshift deletion | exon2 & exon3 & exon4 | nonsynonymous SNV | 229 |
| *BnaA09G0696200ZS* | exon1 | frameshift insertion | exon1 & exon2 & exon4 | nonsynonymous SNV | 11 |
| *BnaA09G0696300ZS* | exon1 | frameshift insertion | exon1 & exon2 & exon3 | nonsynonymous SNV | 14 |
| *BnaA09G0696400ZS* | - | - | exon1 & exon2 & exon3 & exon4& exon5 & exon6 &exon9 & exon11 & exon12 & exon13 | nonsynonymous SNV | 6 |

**Table S****5. Primer sequences of eight markers tightly linked with cuticle wax which exhibit polymorphisms both in the parents (‘4074’ and ‘4075’) and F_2_ population**

| **Markers** | **Physical position on ChrA09 (bp)** | **Primer sequences (5'-3')** |
| --- | --- | --- |
| 9AS190 | 57126447-57126461 | Forward: TTTTAGAACGGAGCGTAAAG  Reverse: AGGATTTGGTGGAGAAGAA |
| *9AS317* | 63727169-63727180 | Forward: TTCTAAAAATGGCTAGGCTG  Reverse: AGTTCTAAACCCCTCCAAAA |
| *9AS323* | 63960763-63960774 | Forward: GCATGCTCGATATTACCTGT  Reverse: TTGTTTGGTTTCAGATCCTC |
| 9AS251 | 64087545-64087558 | Forward: TAAAGGCTCTGCTTCTTCTG  Reverse: TTACACTTACCCTTTGGAGC |
| *9AS329* | 64197882-641979930 | Forward: GAATCAGGTTCGGTCTTAAA  Reverse: GTACGACATTTTCAGTGGGT |
| *9AS334* | 64341728-64341745 | Forward: GGTCAAACATTGAAGAGCAT  Reverse: GTGTGAGGATCCCAAGTTTA |
| 9AS337 | 64453151-64453165 | Forward: CAATCTGAGCATCAAACTGA  Reverse: GTGTGAATTGGTTGTACCCT |
| *9AS339* | 64502145-64502156 | Forward: GACATCATCAAAGGACGAGT  Reverse: TGTATGTCCTGTCTTCCTCC |

**Table S6. Primer sequences used for gene cloning via RT-PCR.**

| **Gene name** | **ZS11 Gene ID** | **Primer sequences (5'-3')** |
| --- | --- | --- |
| *BnaCYP86A4_A09a* | *BnaA09G0695600ZS* | Forward: CACTTCTCTTCTCAATCACCC  Reverse: ACCATCATCCATCAAACG |
| *BnaCYP86A4_A09b* | *BnaA09G0717100ZS* | Forward: GCTGCTTACCAAATCCTCAA  Reverse: TGCTTACGGTCATCCATCA |
| *BnaCYP86A4_A10* | *BnaA10G0004100ZS* | Forward: GCACACCACCACTTTACCAC  Reverse: CATACCGAACAGAAACCACAGT |
| *BnaCYP86A4_C05* | *BnaC05G0006000ZS* | Forward: CCCGATTTGTATCCGACTTA  Reverse: GCATCACATTGGACATTTCC |
| *BnaCER1* | *BnaA09G0721400ZS* | Forward: GGCTACGAAACCAGGCATCCT  Reverse: GAAGTGATGTGGAAGCAGCAG |
| *proBnaCYP86A4_A09a* | *BnaA09G0695600ZS* | Forward: TTTGAAGTCCAACCTGGC  Reverse: TCAAGATTCCTCGGGTCA |
| *proBnaCYP86A4_A09b* | *BnaA09G0717100ZS* | Forward:  tggctgcaggtcgacggatccCTTTGGATTTTAAAAAAACATGTTGTAC  Reverse: tcttagaattcccggggatccATAGCTCTTTGATTAATATGAATGTGGTG |
| *proBnaCYP86A4_A10* | *BnaA10G0004100ZS* | Forward: tggctgcaggtcgacggatccTTTTATAGGGGTTAATACATAGATTTTGAG  Reverse:  tcttagaattcccggggatccATATAGCTCTTTGGTTGGATGCAG |
| *proBnaCYP86A4_C05* | *BnaC05G0006000ZS* | Forward: tggctgcaggtcgacggatccCCGAATCCGACCCGAAAA  Reverse:  tcttagaattcccggggatccTTATAGCTCTTTGGTTGGATGCAG |

**Table S7. Primer sequences used for qRT-PCR analysis.**

| **Gene name** | **ZS11 Gene ID** | **Primer sequences (5'-3')** |
| --- | --- | --- |
| *BnaEPD* | *BnaA09G0695200ZS* | Forward: TTGACAAAGACCACCACG  Reverse: ACCAATGCCTACACCAAGA |
| *BnaAKT* | *BnaA09G0695300ZS* | Forward: ATAGGGAAGGTGGAGCACA  Reverse: TTCACACAGCCCATTCGT |
| *BnaFRO2* | *BnaA09G0695400ZS* | Forward: CATCAATCCTCGGACCAA  Reverse: CTGTAGGAGCGATTCTTGTG |
| *BnaCYP86A4_A09a* | *BnaA09G0695600ZS* | Forward: GCGTACAGCGACACTTTCCT  Reverse: CTCGTCAAAATTTAAAGGCTCCTGA |
| *BnaCYP86A4_A09b* | *BnaA09G0717100ZS* | Forward: CGGAAAGGACACGAAAACC  Reverse: TGGAGGAAAGTGTCGCTGT |
| *BnaCYP86A4_A10* | *BnaA10G0004100ZS* | Forward: TGAAGAAATGGTTAAGACTCGG  Reverse: CTGGGCTCCACTTTCTTGC |
| *BnaCYP86A4_C05* | *BnaC05G0006000ZS* | Forward: TAAAGAAATGGCTAGGACTCGG  Reverse: CTGGGCTCCACTTTCTTGC |
| *BnaGPAT4* | *BnaA09G0695700ZS* | Forward: TGCATTTTGTTCATTCTCATCTCT  Reverse: TCATGTTGGCCTGAACTGCT |
| *BnaPIP1-3* | *BnaA09G0695800ZS* | Forward: AAGGACTACCGACTACAAGGAG  Reverse: TGTTTGGTGCTCTCACGA |
| *BnaPI-GlcNAc* | *BnaA09G0696200ZS* | Forward: GCAAGAGCAGGTGAAGCAA  Reverse: CGTCAACGAAGTTCTCACTACT |
| *—* | *BnaA09G0696300ZS* | Forward: CCGCAACTCCTACTTCTTCT  Reverse: CCACCTTCAGATTCCCTTC |
| *BnaLRAT* | *BnaA09G0696400ZS* | Forward: GCGAAAGGAGACGGTTTA  Reverse: AGACATAGAGAGCCAGCGA |
| *Bna20ox* | *BnaA02G0024900ZS* | Forward: CTCAATCAGTCGTGGTCAAT  Reverse: TGTCTCCTTTCGGGCATA |
| *Bna3ox* | *BnaA06G0105000ZS* | Forward: AATGTGGTCGGAAGGTTTC  Reverse: TCAACGATGTCGCAGTAGTT |
| *DELLA* | *BnaC02G0205300ZS* | Forward: TGACGATGGAAACTCGCA  Reverse: CAGAATCTTGGTCGGGTATG |
| *BnaABI5* | *BnaA05G0087100ZS* | Forward: GATAACATAGGAGGACAGTAT  Reverse: CTCAACTACCTTCTCTACC |
| *BnaSnRK2* | *BnaC01G0336800ZS* | Forward: TCTGGAGATTTGGCTACCC  Reverse: GGACTAAAGAGAGTTCGCCC |
| *BnaCYP707A1* | *BnaC01G0124100ZS* | Forward: TGCTGGTGACGAAGTCTCA  Reverse: TGAAGGCACGAAGAACGA |
| *BnaCYP707A2* | *BnaA04G0188100ZS* | Forward: AGTGGTGTTGCTTCAATGG  Reverse: TGTCTCTCCGATGTAAGGC |
| *BnaACTIN7* | *BnaC02G0037200ZS* | Forward: TATCCTCCGTCTCGATCTCGC  Reverse: CTTAGCCGTCTCCAGCTCTTG |

**Reference**

Fich, E. A., Segerson, N. A., & Rose, J. K. (2016). The plant polyester cutin: biosynthesis, structure, and biological roles. *Annu. Rev. Plant Biol.* 67, 207–233. doi:10.1146/annurev-arplant-043015-111929

Pu, Y., Gao, J., Guo, Y., Liu, T., Zhu, L., Xu. P., et al. (2013). A novel dominant glossy mutation causes suppression of wax biosynthesis pathway and deficiency of cuticular wax in *Brassica napus*. *BMC Plant Biol*. 13, 215. doi:10.1186/1471-2229-13-215

Wen, H., Wang, Y., Wu, B., Feng, Y., Dang, Y., Yang, B., et al. (2021). Analysis of Wheat wax regulation mechanism by liposome and transcriptome. *Front. Genet*. 12, 757920.  doi:10.3389/fgene.2021.757920
